# Morphomigrational description as a new approach connecting cell's migration with its morphology

**DOI:** 10.1038/s41598-023-35827-9

**Published:** 2023-07-07

**Authors:** Tomasz Kołodziej, Aleksandra Mielnicka, Daniel Dziob, Anna Katarzyna Chojnacka, Mateusz Rawski, Jan Mazurkiewicz, Zenon Rajfur

**Affiliations:** 1 Department of Pharmaceutical Biophysics, Faculty of Pharmacy, Jagiellonian University Medical College, ul. Medyczna 9, 30-688 Kraków, Poland; 2 Department of Molecular and Interfacial Biophysics, Faculty of Physics, Astronomy and Applied Computer Science, Jagiellonian University, ul. Lojasiewicza 11, 30-348 Kraków, Poland; 3 BRAINCITY, Laboratory of Neurobiology, The Nencki Institute of Experimental Biology, PAS, ul. Ludwika Pasteura 3, 02-093 Warsaw, Poland; 4 Cellular Signalling and Cytoskeletal Function Laboratory, The Francis Crick Institute, 1 Midland Road, NW1 1AT London, United Kingdom; 5 Laboratory of Inland Fisheries and Aquaculture, Department of Zoology, Faculty of Veterinary Medicine and Animal Science, Poznań University of Life Sciences, ul. Wojska Polskiego 71C, 60-625 Poznań, Poland; 6 Jagiellonian Center of Biomedical Imaging, Jagiellonian University, 30-348 Kraków, Poland

**Keywords:** Cell migration, Biophysics

## Abstract

The examination of morphology and migration of cells plays substantial role in understanding the cellular behaviour, being described by plethora of quantitative parameters and models. These descriptions, however, treat cell migration and morphology as independent properties of temporal cell state, while not taking into account their strong interdependence in adherent cells. Here we present the new and simple mathematical parameter called *signed morphomigrational angle* (*sMM angle*) that links cell geometry with translocation of cell centroid, considering them as one morphomigrational behaviour. The sMM angle combined with pre-existing quantitative parameters enabled us to build a new tool called *morphomigrational description*, used to assign the numerical values to several cellular behaviours. Thus, the cellular activities that until now were characterized using verbal description or by complex mathematical models, are described here by a set of numbers. Our tool can be further used in automatic analysis of cell populations as well as in studies focused on cellular response to environmental directional signals.

## Introduction

Cell migration is one of the most significant phenomena present in living organisms, being involved in both physiological and pathological processes^1^. It requires a very precise coordination of various intracellular biochemical pathways^2^, which are regulated by interactions with the physicochemical microenvironment^3–5^. Those biochemical processes are reflected in migration mode and cell shape as interdependent properties of cellular behaviour^6,7^. One of the most popular techniques used in quantitative studies of cell migration is wide-field microscopy combined with time-lapse imaging.

To quantify cell movement, one can calculate instantaneous velocity, turning angle, or just cell displacement between two consecutive frames^8–11^. These values can be first averaged (or analysed in any other way) for each cell separately and then summed up to investigate cell population (‘cell-based parameters’) or gathered to draw conclusions from all available time steps, regardless of which specific cell track the time steps belong to (‘step-based parameters’)^12^. Alternative parameters describe cell movement based on a particular cell track. The Mean-Squared Displacement (MSD) values can be calculated to characterize whether the cell is moving in diffusive, sub-diffusive or ballistic manner^13^. In addition, the persistent random walk model, gives information about parameters such as persistence time that indicates the average time between significant changes in movement direction, or random motility coefficient which is analogous to diffusion coefficient^14,15^. Just from the analysis of displacements, it is possible to calculate the confinement index^12^ that characterizes the movement effectiveness, as the well as cumulative (also called accumulated^16^) distance^9^ which is a sum of all displacements, indicating how far the cell migrated. There are several quantitative parameters oriented on movement directionality as well, for example: auto-correlation function of cell velocities to describe how directional cell movement is^17^, directionality time describing the time scale above which migration path orientation remains correlated due to the external cue^10^, or individual moment of inertia tensor that shows preferred migration direction on defined plane^18^. Those are only a few of many quantitative movement metrics that describe how an individual cell or a cell population is moving.

Since cell migration and morphology are mutually dependent, the accurate description of cell shape is equally important in analysis of cellular behaviour. Most popular quantitative shape descriptors have been reviewed in several works^19–21^. To compare how spread cells are, one can calculate their area and perimeter^19^. The shape can also be described by general parameters, such as: elongation^22^ normalized between 0 (rounded shape) and 1 (infinitely elongated ellipse); circularity^23^ (also called by compactness^24^) which describes to which extent the shape is similar to a perfect circle; or aspect ratio of minimal to maximal dimension^25,26^. The more specific parameters were also constructed to quantify the regularity of a shape. For example, the solidity parameter^27^ informs us whether the cell has holes or irregular boundaries while the increase in the value of the dispersion parameter from 0 to 1 is indicative of cell shape becoming less smooth^28^. Cell morphology can also be related to its surroundings or external signals, for example by measuring the angle between the main axis of shape and external signals^29,30^, or comparing orientation of the cell nucleus to the orientation of the whole cell^25^. Nevertheless, those parameters are calculated for static images, and they do not inform about dynamics of a particular cell. There are several approaches of dynamic quantification of shape, such as DECCA^31^ describing the cell motion over time, linking cell motility and shape, TSRVF-PCA with the VAR model to investigate temporal evolution of shapes^32^, as well as evolution of Fourier shape descriptors^4^. Those methods, however, are rather focused solely on quantifying dynamics of cell shape and were not designed to analyse it in the context of cellular displacement direction.

**Figure 1:**
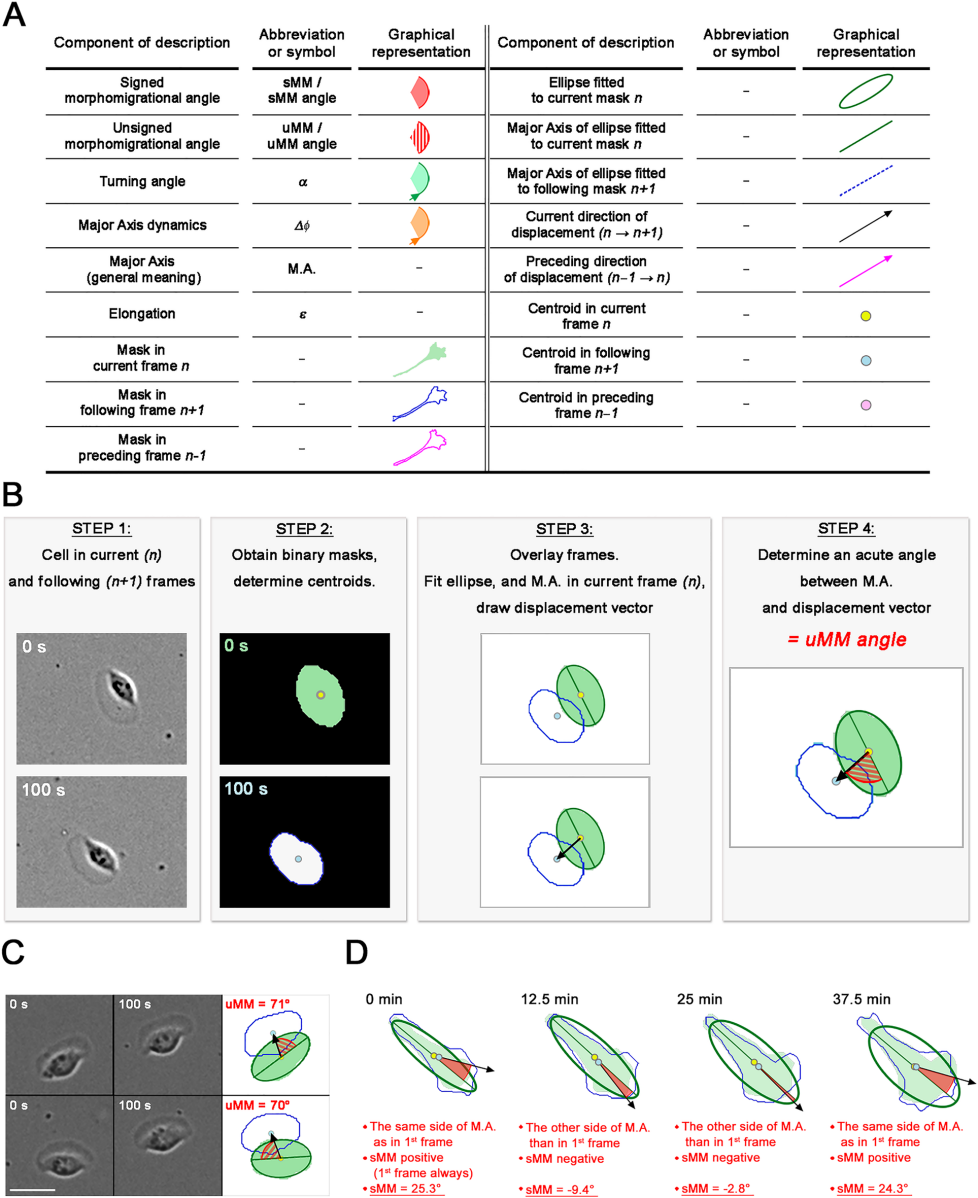
Graphical representation of unsigned and signed morphomigrational angles with legend. Green masks and blue outlines represent currently analysed shape and following shape, respectively. Black arrow signifies movement direction (magnified displacement vector), green line is major axis of an ellipse fitted to current shape (green ellipse). Yellow and blue circles mark shape centroids in current (n) and following (n + 1) frame respectively. Red area with stripes marks uMM angle, red solid area marks sMM angle. Scale bars represent 20 µm. (**A**) Terminology together with abbreviations, symbols and their graphical representations. (**B**) Procedure of uMM angle calculation (see: Materials and Methods). (**C**) Migrating fish keratinocyte described by uMM angle. Values of uMM angle are very similar, regardless of the angle is constructed on the right or the left side of displacement vector (top and the bottom row, respectively, see: Materials and Methods). (**D**) Changing sign of sMM angle for migrating MEF 3T3 cell. sMM angle is positive while being constructed on the same side of M.A. as in first frame and negative being constructed on the opposite side (see: Materials and Methods).

Nonetheless, since every asymmetric modification of cell shape results in displacement of cell centroid, there are no clear criteria to distinguish between the “just” change of shape from cell movement in the bright field microscopy. Cell motility is thus tightly connected to the cell membrane dynamics^33,34^. Even if previous work aimed to disentangle shape and movement by finding processes dependent on cell speed and membrane dynamics separately^35^, the interdependence of cell movement and shape remains indisputable. Here, we propose to recognize cell morphology and migration as the one amalgamated *morphomigrational behaviour* and to analyse them jointly under the name of *morphomigrational description*. This description consists on assigning four quantitative parameters (called here *the building blocks*) to each frame of investigated cell, to uniquely describe cellular dynamics. The first building block we introduce here, is the novel and simple geometrical metrics called *signed morphomigrational angle* (*sMM angle* or *sMM*) which is an acute angle measured between major axis of the ellipse fitted to cell shape and displacement vector between two frames. It represents the movement direction of a centroid in the context of the cell orientation on 2D plane, as shown in Fig. 1 B–D. To our knowledge it is a first such descriptor that links cellular movement with the shape of the cell. Second building block represents the dynamics of major axis (M.A. dynamics) between two consecutive frames, characterising the rearrangement of cell shape on 2D plane. Two remaining building blocks of morphomigrational description are constituted by already known, existing parameters, i.e. turning angle between two consecutive displacement vectors and elongation of the cell.

This work describes the calculation method, advantages and already identified limitations of morphomigrational description. The potential application of this method is demonstrated by quantitative description of several cellular behaviours, such as perpendicular migration, lateral migration, lateral and perpendicular stretching, lateral turn back or contraction of the rear protrusion, that were observed for fish keratinocyte, MEF3T3 fibroblast and HEK293 epithelial cell. This approach allowed us to assign quantitative values of morphomigrational description to the mentioned behaviours, showing how they can be identified by potential automatic analyses.

## Results

Here we present the building blocks of morphomigrational description and provide practical examples of their application in an analysis of migrating cells. First, we show the rationale for choosing those descriptors with explanation how do they complement each other in describing the cell behaviour. Then, we apply the new method to quantify several cellular morphomigrational behaviours, e.g. different types of directional migration, stretching and rear retraction, observed for three individual exemplary cells: fish epithelial keratinocyte, MEF3T3 fibroblast and HEK293 epithelial cell which shape and movement differ from each other significantly. The terminology used in this work, together with abbreviations, symbols and their graphical representations, are shown in Fig. 1A.

### Combination of sMM angle and M.A. dynamics with turning angle and shape elongation—rationale and examples

**Figure 2:**
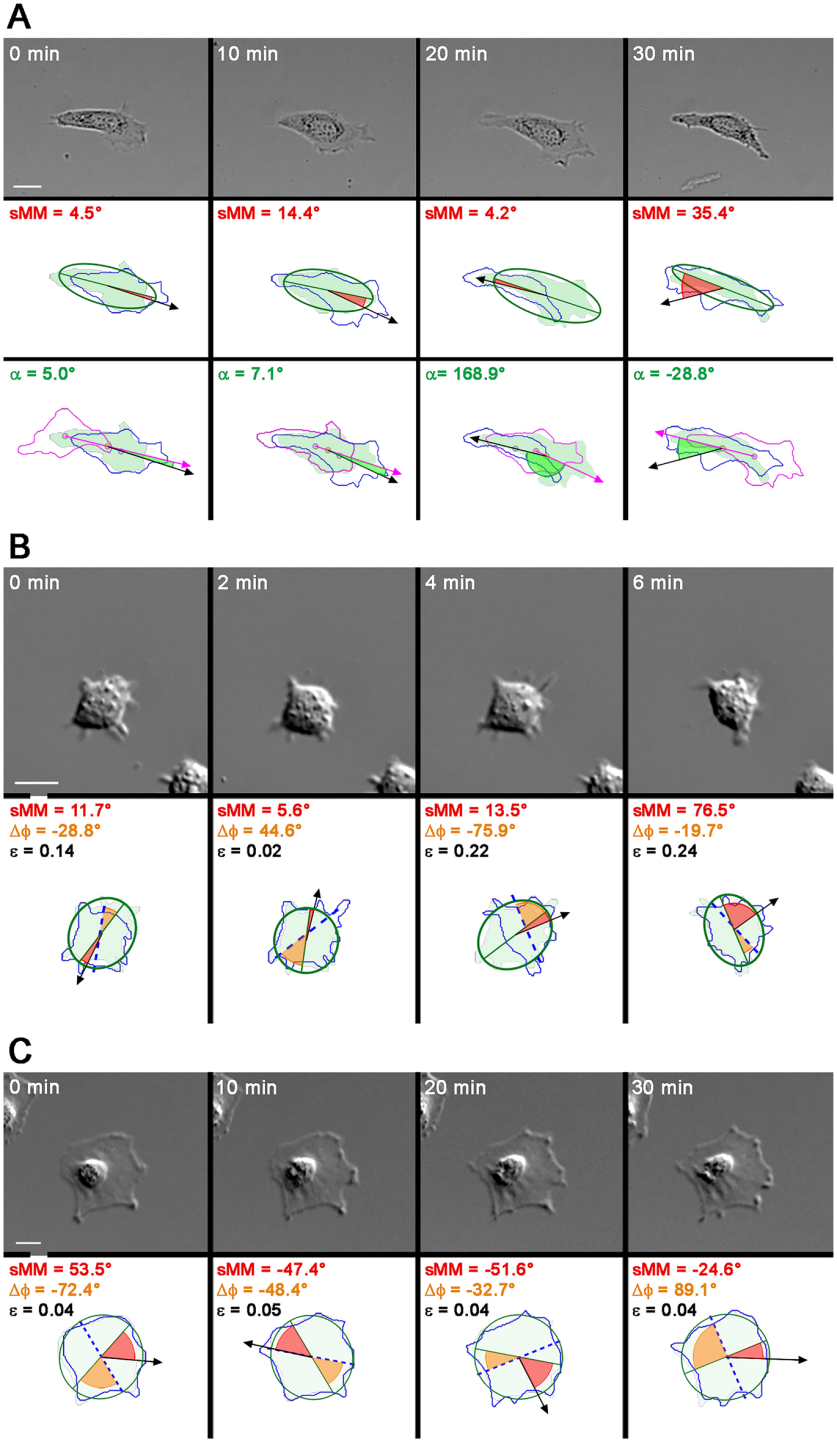
Complementary roles of building blocks of morphomigrational description and limitation of sMM angle. Magenta outlines, green masks, and blue outlines represent previous (n − 1), current (n) and following (n + 1) frame, respectively. Magenta and black arrow mark previous and current movement direction (resized and elongated displacement vectors), green line is major axis of ellipse fitted to current shape (green ellipse) and dashed blue line shows major axis of an ellipse fitted to shape in following frame. Yellow, blue and pink circles mark shape centroids in current (n), following (n + 1) and preceding (n − 1) frames, respectively. Solid red and green areas mark sMM angle and turning angle, respectively. Orange solid area marks M.A. dynamics (Δϕ) defined as an angle between current and following major axes; ε denotes shape elongation. Scale bar represents 20 µm. (**A**) Comparison of sMM and turning angles of migrating MEF 3T3 cell. The complementary roles of sMM and turning angle is especially visible in 3rd frame (20 min) when cell turns back along the major axis, exhibiting low sMM angle and high turning angle, enriching the morphomigrational description. (**B**) Combination of sMM angle and M.A. dynamics in description of HEK 293 cellular behaviour in consecutive time frames. Large value of M.A. dynamics in second frame (2 min) comes mostly from stretching the shape, while in 3rd frame (4 min) from the tail retraction that causes significant rearrangement of cell shape. The rearrangement is also visible in sMM angles since in 3rd frame (4 min) cell migrates rather laterally to the major axis, while in the 4th frame (6 min) migrates perpendicularly to it. (**C**) Quasi-polygonal MEF 3T3 cell in consecutive time frames. The regularity of cell’s shape is reflected in low elongation of fitted ellipse. Since the ellipse is very similar to the circle and major axis orients itself rather randomly, the sMM angle is not much informative and morphomigrational description should be based on remaining building blocks.

Figure 2A shows the complementary functions of the sMM angle and the turning angle (α) in the morphomigrational description. Although they seem to be very similar, their origin is substantially different. sMM angle is constructed on major axis and displacement vector (2nd row) while turning angle is constructed on two consecutive displacement vectors (3rd row). It is reflected in different values of both angles in particular situations. In Fig. 2A MEF 3T3 starts from directional migration (0–20 min.) described by low turning angles, which is followed by direction reversal between 20 and 30 min, described by incidentally very high value of turning angle. During the whole sequence this cell migrates along its major axis, which is described by low and very low values of the sMM angle. In this example, the combination of sMM and turning angle directly links migration direction and cell shape to accurately describe morphomigrational behaviour of the cell, including direction reversal.

Since sMM angle is constructed along a major axis of an ellipse fitted to shape, the consecutive positions of major axis in time (here defined as M.A. dynamics) are crucial in morphomigrational description. In Fig. 2B high M.A. dynamics of HEK293 cell comes mostly from remodelling cellular morphology in consecutive frames. In the beginning, the cell shape became rounder (0–2 min), which is visible in large M.A. dynamics and low elongation. The cell then (2–4 min) stretched itself by creating new protrusion to finally contract and translocate the cell body (4–6 min). The latter behaviour is quantitively described by a low value of sMM angle (sMM = 13.5°) at time point 4 min, indicating the movement along M.A. paired with high M.A. dynamics (*Δϕ* = − 75.9°) describing the fact that cell geometry aligns in almost perpendicular direction in the next frame. In last frame (6th min) high value of sMM angle (sMM = 76.5°) indicates that cell moves perpendicularly to its M.A., but the shape remains stable (*Δϕ* = − 19.7°). On the other hand, Fig. 2C presents a well-spread and slowly migrating MEF 3T3 fibroblast. Its shape is quite regular, therefore it can easily be approximated to the circle (ε = 0.04–0.05) in all of the analysed frames. Such shape imposes almost random orientation of the fitted ellipse which is indicated by moderate to very high M.A. dynamics and causing random values of sMM angle. In such case, morphomigrational description benefits more from quantification of turning angles, M.A. dynamics, and elongation, while random values of sMM angle just highlight the circularity of cell shape.

### Morphomigrational description of cellular behaviours

To build morphomigrational description, we first visually assess cellular behaviour and then calculate values of their quantitative descriptors. To make the analysis of quantitative parameters more intuitive, their exact values were grouped in coarse-grain ranges (described as *very low, low, moderate, high, very high*) presented in Table 1. Exemplary morphomigrational behaviours exhibited by model cells within shown timeframes are marked by numbers and letters (K1—keratinocyte, M1–M5—MEF 3T3 cell, H1–H5—HEK 293 cell) and exact values of their morphomigrational parameters are presented in Sup. Table S7. We subsequently analyse those behaviours in Tables 2, 3 using coarse-grain description, to illustrate how do they quantitatively describe these cellular actions. Some presented fragments (H1, M2, M3 and M5) represent several morphomigrational behaviours while rest of them (K1, H2–H5, M1 and M4) represent only single type of event. Visual representation of all four building blocks of morphomigrational description in each time point are shown in Sup. Figs. S3–S6.

**Table 1:** Definitions of coarse-grain descriptions of quantitative parameters used in this work.

Parameter	Values		Coarse-grain description	Parameter	Values		Coarse-grain description
	From	To			From	To	
sMM angle (sMM)	− 15° ≤	≤ 15°	sMM very low	M.A. dynamics (Δϕ)	− 10° ≤	≤ 10°	Δϕ very low
	− 45° ≤	< − 15°	sMM low		− 20° ≤	< − 10°	Δϕ low
	15° <	≤ 45°			10° <	≤ 20°	
	− 70° ≤	< − 45°	sMM moderate		− 45° ≤	< − 20°	Δϕ moderate
	45° <	≤ 70°			20° <	≤ 45°	
	− 90° ≤	< − 70°	sMM high		− 60° ≤	< − 45°	Δϕ high
	70° <	≤ 90°			45° <	≤ 60°	
Turning angle (α)	− 60° ≤	≤ 60°	α low		− 90° ≤	< − 60°	Δϕ very high
	− 90° ≤	< − 60°	α moderate		60° <	≤ 90°	
	60° <	≤ 90°		Elongation (ε)	0 ≤	≤ 0.1	ε low
	− 135° ≤	< − 90°	α high		0.1 <	≤ 0.6	ε moderate
	90° <	≤ 135°			0.6 <	≤ 1.0	ε high
	− 180° ≤	< − 135°	α very high				
	135° <	≤ 180°

**Table 2:** Morphomigrational description of fragments K1, H1–H5.

Fragment of sequence	Parameter	Coarse-grain description of numerical values	What does the quantitative parameter inform about	Morphomigrational behaviour
K1	sMM	Moderate and high, sign does not change	Displacement perpendicular to major axis, always on the same side of it	Perpendicular displacement [frames 16–19] and askew displacement [frame 20]
	α	Low, sign might change	Movement in particular direction. Changing sign informs about no visible bias in the clockwise nor anti-clockwise direction	
	Δϕ	Very low, sign might change	No rapid changes of cell shape	
	ε	Moderate, increasing	Moderately elongated	
H1	sMM	Very low and low, sign does not change	Displacement along the major axis	Lateral displacement [frames 3, 5, 6]
	α	Low, sign might not change	Movement in particular direction. Changing sign informs about no visible bias in the clockwise nor anti-clockwise direction	
	Δϕ	Very low and low, sign might not change	Cell shape rotates slightly anti-clockwise	
	ε	Moderate, constant	Moderately elongated cell	
	sMM	Very low and low, the same sign	Displacement along the major axis	With occasional lateral U-turns [frames 4, 7]
	α	Low → high/very high, sign might change	Cell performs the turn back	
	Δϕ	Very low and low, the same sign	Barely noticeable anti-clockwise turn of the shape	
	ε	Moderate, constant	Moderately elongated cell	
H2	sMM	Moderate and low, changing sign	Displacement in various directions regarding M.A.	Rounded cell with chaotic movement [frames 12–14]
	α	High and very high, changing sign	Nondirectional movement	
	Δϕ	Low, moderate and high, the same sign	Shape changes its arrangement in clockwise direction	
	ε	Moderate and low, decreasing	Rounded or regular polygonal shape	
H3	sMM	Very low → high, the same sign	Rapid change of sMM from lateral to perpendicular movement	Rear protrusion retraction with directional displacement. Cell geometry changes from parallel to perpendicular to movement direction [frames 22–23]
	α	Low, changing sign	Movement in particular direction. Changing sign informs about no visible bias in the clockwise nor anti-clockwise direction	
	Δϕ	High → low, the same sign	Incidental rotation of cell shape, further conservation of this arrangement on 2D plane	
	ε	Moderate, stable	Moderately elongated cell	
H4	sMM	Moderate and high, changing sign	Protrusions are created perpendicularly and askew to M.A. on both sides of it. The same sign in the end of sequence indicates perpendicular displacement	Perpendicular/askew stretching ended with perpendicular displacement [frames 24–28]
	α	Very high, moderate and low sign does not change	Chaotic movement ended by directional movement	
	Δϕ	Very low and low, changing sign	Shape slightly changes its arrangement first anti-clockwise and then clockwise	
	ε	Moderate, increasing	Cell slightly stretches itself while still being moderately elongated	
H5	sMM	Moderate, the same sign	Displacement askew to the major axis constantly on the same side of M.A.	Lateral stretching with perpendicular displacement [frames 31–34]
	α	High → low, changing sign	Cell first changes movement direction (high α) and then stabilizes it (low α)	
	Δϕ	Low and very low, mostly the same sign	Cell arrangement slightly changes in the beginning and stabilizes itself in next frames	
	ε	Moderate, increasing	Cell stretches itself

**Table 3:** Morphomigrational description of selected fragments M1–M5.

Fragment of sequence	Parameter	Coarse-grain description of numerical values	What does the quantitative parameter inform about	Morphomigrational behaviour
M1	sMM	Very low and low, sign does not change	Displacement along the major axis, lamellipodium created mostly on one side of M.A.	Lateral displacement [frames 2–5]
	α	Low, sign might change	Movement in particular direction. Changing sign informs about no visible bias in the clockwise nor anti-clockwise direction	
	Δϕ	Very low, sign might change	No rapid changes of cell shape	
	ε	High and moderate, decreasing	Highly elongated cell that slightly shrinks	
M2	sMM	Very low and low, sign does not change	Displacement along the major axis, lamellipodium created mostly on one side of M.A.	Lateral displacement [frames 17–18 and 20–21]
	α	Low, sign might change	Movement in particular direction. Changing sign informs about no visible bias in the clockwise nor anti-clockwise direction	
	Δϕ	Very low, sign might change	No rapid changes of cell shape	
	ε	High, constant	Highly elongated cell	
	sMM	Very low and low, sign might change	Displacement along the major axis	With lateral U-turn [frame 19]
	α	Low → very high → low, sign might change	Cell performs the turn back	
	Δϕ	Very low, sign might change	No rapid changes of cell shape	
	ε	High, constant	Highly elongated cell	
M3	sMM	Low → high, sign does not change	Incidental displacement of centroid perpendicular to M.A. indicates perpendicular protrusion, roughly in the cell centre	Lateral displacement with creation of small perpendicular protrusion in central part [frames 22–23]
	α	Low, sign does not change	Displacement turned anti-clockwise according to the new protrusion on side of the cell	
	Δϕ	Very low, sign might change	No rapid changes of cell shape	
	ε	High, constant	Highly elongated cell	
	sMM	High → very low, sign might change	Protrusion was not sustained, cell continued the previous lateral displacement	Followed by movement lateral to major axis [frames 24–25]
	α	Moderate → low, sign changes	Cell goes back from creating perpendicular protrusion to previous stable directional movement	
	Δϕ	Very low, sign might change	No rapid changes of cell shape	
	ε	High, constant	Highly elongated cell	
M4	sMM	Low and moderate, the same sign	Cell creates protrusion on one side of M.A. (the same sign), while moving laterally	Lateral displacement with lamellipodium on one side of M.A. [frames 35–38]
	α	Low, sign might change	Movement in particular direction. Changing sign informs about no visible bias in the clockwise nor anti-clockwise direction	
	Δϕ	Very low, the same sign	Cell shape rotates slightly clockwise	
	ε	High, increasing	Highly elongated shape, still elongating itself	
M5	sMM	Very low and low, sign does not change	Displacement along the major axis	Lateral displacement with lateral U-turn [frames 39–40]
	α	Low → very high, sign might change	Cell performs the turn back	
	Δϕ	Very low, the same sign	Cell shape rotates slightly clockwise	
	ε	High, increasing	Highly elongated shape, still elongating itself	
	sMM	Moderate, likely the same sign	Cell slowly creates dominant protrusion on one side of M.A. and on one of the cells’ end	Followed by slow change of direction (restoration of lamellipodium in previous direction) [frames 41–42]
	α	Low and moderate, likely the same sign	Slow change of displacement direction	
	Δϕ	Very low, the same sign	Cell shape rotates slightly clockwise	
	ε	High, increasing	Highly elongated shape	
	sMM	Very low and low, sign does not change	Displacement along the major axis	And further lateral stretching (several direction reversals along major axis) followed by directional migration [frames 43–45]
	α	High → high → low, sign might change	Two turn-backs (lateral stretching) followed by lateral movement	
	Δϕ	Very low, sign changes	Stabilization of previous shape rotation	
	ε	High, increasing	Highly elongated shape, cell stretches itself	

**Figure 3:**
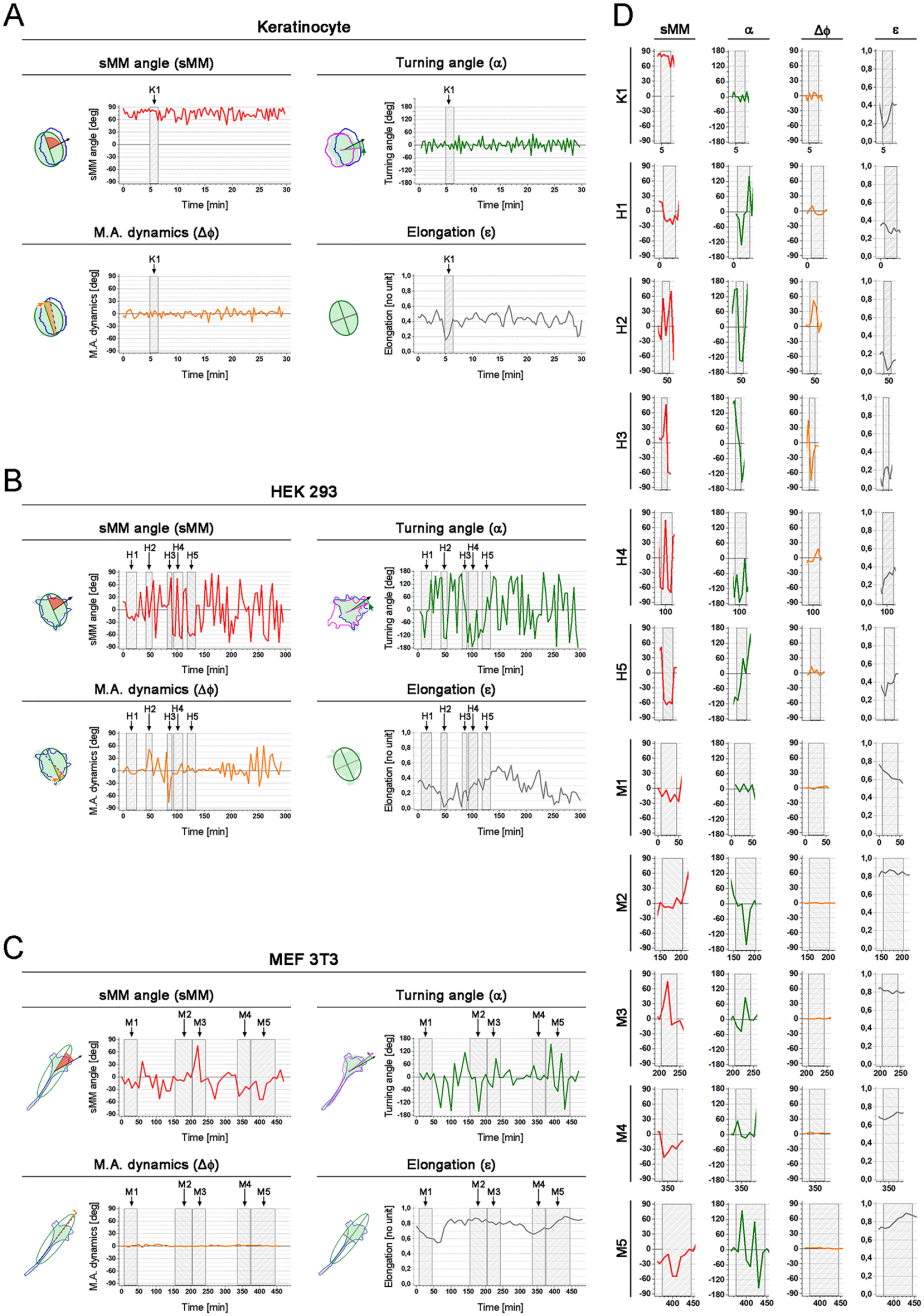
Time evolution of morphomigrational parameters. (**A**) Time sequences of sMM angle, turning angle, M.A. dynamics and elongation calculated for single keratinocyte. Fragment K1 for each parameter is analysed in Table 2. (**B**) Time sequences of sMM angle, turning angle, M.A. dynamics and elongation calculated for single HEK293 epithelial cell. Fragments H1-H5 for each parameter are analysed in Table 2. (**C**) Time sequences of sMM angle, turning angle, M.A. dynamics and elongation calculated for single MEF 3T3 fibroblast. Fragments M1-M5 for each parameter are analysed in Table 3. (**D**) Zoomed fragments of analysed fragments: K1, H1–H1 and M1–M5.

The movement perpendicular to major axis of the cell is described by moderate and high sMM angles of the same sign, accompanied by low values of turning angle that is visible in K1 behaviour of keratinocyte (Fig. 3A, Table 2, Sup. Fig. S3). The moderate value of sMM with no significant change in turning angles in the end of K1 sequence indicate the fact that keratinocyte extended its lamellipodium slightly sideways, although it did not influence the direction of migration. Thus, we can see that morphomigrational description is sensitive for the shape change. The temporal phase of perpendicular migration can also be seen in the end of H4 sequence (Fig. 3B, Table 2, Sup. Fig. S4) in which two last frames are represented by moderate and high sMM angle of constant sign. The turning angle represents initial change of displacement direction (very high α) that is further maintained (low α).

The perpendicular and askew stretching, a more chaotic movement, is also indicated by high and moderate sMM angles, but the sign changes as protrusions are created on different sides of the cell. This behaviour is observed in four initial frames of H4 sequence (Fig. 3 B, Table 2, Sup. Fig. S4), being also indicated by moderate, high and very high turning angles. Very low and low M.A. dynamics illustrate only minimal rotation of cell shape, while increasing elongation (from 0.1 to 0.34) indicates that the creation of perpendicular and askew protrusions results in cell elongation.

On the other hand, significantly different morphomigrational description is provided for lateral migration, being illustrated by low sMM angle and very low and low turning angles, visible in M1, M4, as well as fragments of H1 sequence (Fig. 3B,C, Tables 2, 3, Sup. Figs. S3, S4 and S6). Generally, the same sign of sMM angle suggests that leading edge is created asymmetrically, on one side of major axis, while changing sign and low values of sMM would indicate protrusions created symmetrically to major axis. The sign of turning angle may change, although it is not necessary. The shape alignment on 2D plane is described by M.A. dynamics which low values and changing sign clearly indicate that in M1 fragment MEF3T3 cell exhibits persistent stable shape, while the same cell in fragment M4 slightly rotates due to the asymmetrically created lamellipodium.

In the absence of directional signals, many types of cells can rapidly change their direction. Here we have the opportunity to observe the lateral U-turns, i.e. when cells suddenly start moving in the opposite direction, while still migrating along their major axes. The lateral U-turn is represented by constantly low sMM angles and incidental increase of turning angle followed by its rapid decrease. Such morphomigrational behaviour can be observed in M2 sequence, at the beginning of M5 and fragments of H1 sequence (Fig. 3B,C, Tables 2, 3, Sup. Figs. S3, S5, S6). On the other hand, the direction change does not need to be performed only during the one time point, since it can be caused by a slow restoration of lamellipodium on one side of the cell. In the middle of M5 sequence (400–410 min) we can observe moderate sMM angles of the same sign paired with low and moderate turning angles that inform about the gradual change of centroid movement.

Beside the directional movements we have also observed the two types of lateral stretching. The simplest one is based on the lateral movement of the centroid that can be observed in the end of M5 sequence (Fig. 3C, Table 3, Sup. Fig. S6). It is indicated by low sMM values that may change their sign and turning angles of high and very high values. Second type of lateral stretching can be observed in H5 fragment of HEK293 cell (Fig. 3B, Table 2, Sup. Fig. S4) in which lateral stretching of the cell relates to perpendicular displacement of cell centroid. It is quantitatively described by moderate sMM angles of the same signs and moderate turning angles of changing signs. In that case, the stretching is accented by the increasing elongation (from 0.25 to maximally 0.4).

The morphomigrational description can also be useful in describing other types of protrusive dynamics. In H3 fragment (Fig. 3B, Table 2, Sup. Fig. S3) we can observe the retraction of cell rear connected with change of cell geometry. It is described by a rapid increase of sMM angle, constantly low turning angle and incidental high value of M.A. dynamics. On the other hand, in M3 fragment (Fig. 3C, Table 3, Sup. Fig. S5) we can observe the short-lived lamellipodium that was created in the centre and was then relocated into the leading edge of fibroblast. This behaviour is reflected in the incidental high value of sMM angle followed by its decrease indicating lateral movement. This relocation of side lamellipodium is also indicated by incidental moderate value followed by low values of turning angles.

In addition to the behaviours outlined so far, we have found that in some instances the cells exhibited chaotic behaviors that were not able to be interpreted using sMM angle. In H2 fragment (Fig. 3B, Table 2, Sup. Fig. S3) the HEK293 cell is rounded (low elongation) causing random orientation of major axis on 2D plane (high M.A. dynamics). Thus, the morphomigrational description of such case is rather focused on turning angles, elongation, and M.A. dynamics, than on the sMM angle.

**Figure 4:**
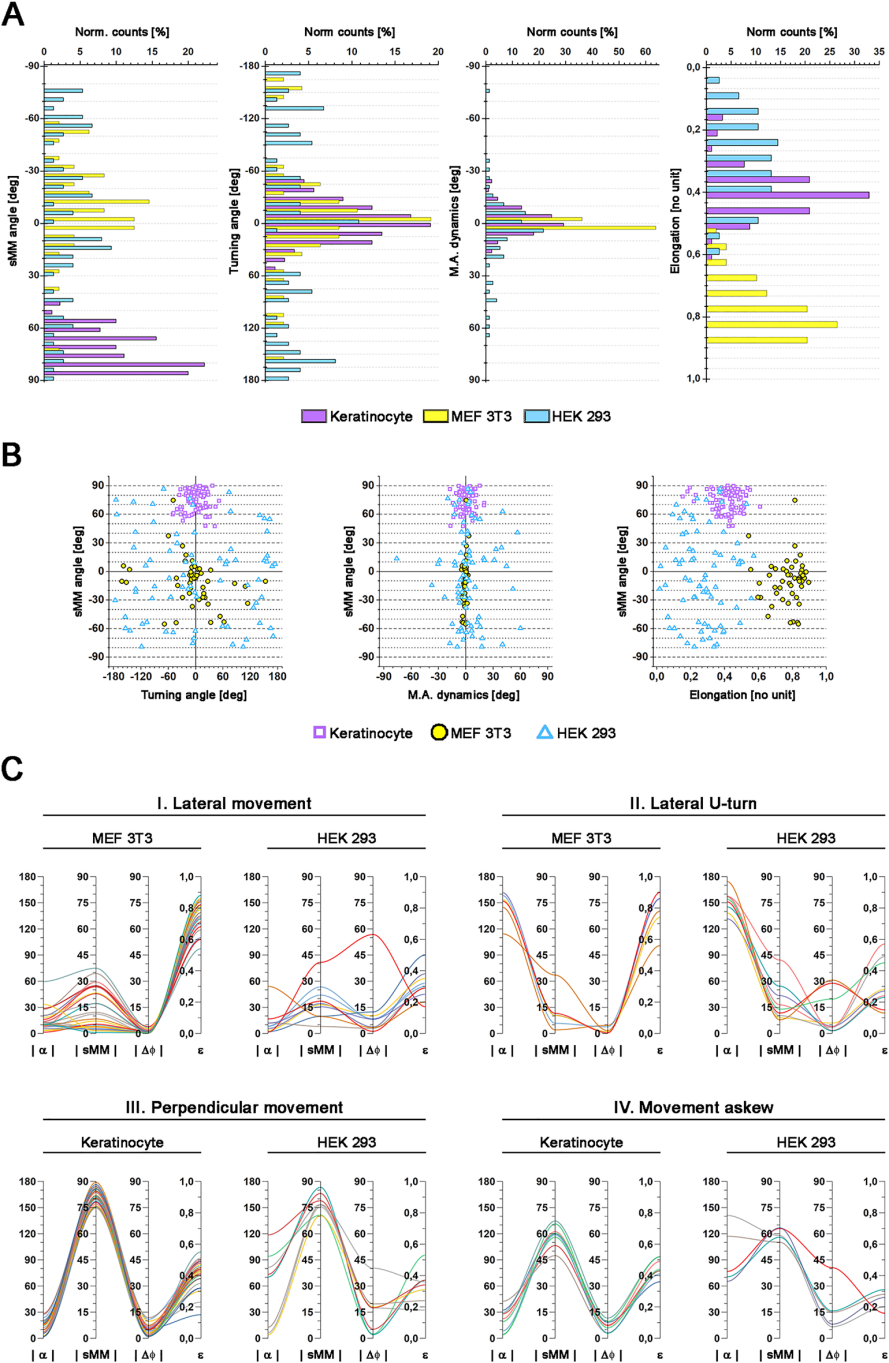
Summative graphs of morphomigrational parameters. (**A**) Histograms of sMM angle, turning angle and M.A. dynamics calculated for three individual exemplary cells: keratinocyte, MEF3T3 cell and HEK293 cell, that were exhibiting different migration modes. Number of frames for each cell is described in section “Image analysis and quantitative descriptors of cell shape and cell migration” of “Materials and methods”. (**B**) sMM angle plotted against turning angle M.A. dynamics and elongation for three individual exemplary cells: analysed keratinocyte, MEF 3T3 and HEK 293. Values of different quantitative descriptors groups differently for each exemplary cell. Number of frames for each cell is described in section “Image analysis and quantitative descriptors of cell shape and cell migration” of “Materials and methods”. (**C**) Different morphomigrational behaviours that were common for two different examined cells, described by cell elongation and absolute values of sMM angle, turning angle, and M.A. dynamics.

The overall behaviour of the cell observed with specific time interval can be considered as the set of its behaviours in time. Thus, the different frequencies with which morphomigrational behaviours occur, as well as their persistence within time sequences, will be different between cells and conditions in which the cells are in. This summative picture of the morphomigrational behaviours within the whole time-lapse sequence are well visible on histograms (Fig. 4A) and patterns created by sMM angle plotted against turning angle, M.A. dynamics and elongation (Fig. 4B, Table 4). They can serve as the visual aid for describing the overall cellular behaviour, showing similarities and differences between cells. However, since every plot presents only some fragments of morphomigrational description, they will be less accurate than analysis of time sequences. Even though, those plots could help in spotting differences as well as similarities between examined cells of different types. For example: the overall behaviour of keratinocyte is characterised by moderate and high sMM values of the same sign and low turning angles, representing persistent movement perpendicular or askew to its major axis. On the other hand, the MEF 3T3 cell migrates laterally, performing some U-turns and stretching, which is represented by low sMM and mostly low turning angles, with incidental high turning angles; while the shape stability is accented by low M.A. dynamics. Largely spread values of sMM angle and turning angles in the HEK293 distributions represent a variety of different morphomigrational behaviours, including lateral and perpendicular stretching, lateral, askew and perpendicular migration. What is interesting, however, is that the shown distributions of M.A. dynamics for keratinocyte and HEK293 cell are not much different, since the latter distribution includes only a few higher values. This means that shape stability in consecutive frames is rather similar for both HEK and keratinocytes, but directional movement perpendicular to major axis specific for keratinocyte (described by the high sMM angles and low turning angles) determines the difference between those two cell types. The deeper understanding of such patterns will need further studies of higher number of cells and classification of their various behaviour. It may thus create a useful tool for characterizing different cell types, by looking at their overall population behaviours.

**Table 4:** Mean values and standard deviation of sMM angles, turning angles, M.A. dynamics and elongations. Number of frames for each cell is described in section “Image analysis and quantitative descriptors of cell shape and cell migration” of “Materials and methods”.

	sMM angle [deg]	Turning angle [deg]	M.A. dynamics [deg]	Elongation [no unit]
Keratinocyte	74.5 ± 10.8	− 0.1 ± 21.6	0.1 ± 7.2	0.42 ± 0.08
MEF 3T3	− 10.4 ± 23.9	− 6.7 ± 62.6	− 0.7 ± 1.6	0.77 ± 0.09
HEK 293	− 4.9 ± 46.4	8.9 ± 114.7	0.4 ± 9.2	0.28 ± 0.14

We also examined how the similar morphomigrational behaviours are described in different cells. Figure 4C shows how four morphomigrational behaviours are illustrated by the parameters comprising morphomigrational description. For clarification, they are described by elongation (ε) and absolute values of turning angle (|α|), sMM angle (|sMM|), M.A. dynamics (|Δϕ|). We selected four examples of morphomigrational behaviours in different cell types to illustrate that those same behaviours have consistent values of the parameters for all cells shown. This is still the case even if the cells have globally different movement and shape dynamics. Each line on the plot represents single frame in which morphomigrational behaviour was observed. The number of any morphomigrational behaviour is different for keratinocyte, HEK293 and MEF3T3 cells, thus the number of lines differs in each plot.

In Fig. 4C I we observe lateral movement for single MEF 3T3 and HEK 293. In both cases, the lateral movement is characterised by low turning angles and low sMM angles, both of which indicate the cells consistently migrating in one direction. They differ however in M.A. dynamics and elongation, with MEF 3T3 cell being more elongated and preserving the same orientation on 2D plane better, than HEK293 cell. Despite the observed similarities, we can see that in one frame of HEK293 cell, continuous lateral movement was coupled with rapid rotation of the cell, as indicated by the high value of M.A. dynamics (|Δϕ| = 56.8°). This clearly illustrates how lateral movement can differ in different cell types. The lateral U-turns (Fig. 4C II) in MEF 3T3 and HEK 293 cells are evidenced by: (a) high and very high turning angles (|α| ≥ 90°), consistent with cells rapidly changing their migration direction and (b) low sMM angles that characterize movement along major axis. As in previous case, the MEF 3T3 and HEK293 cells are different in their M.A. dynamics, illustrating higher shape dynamics exhibited by HEK293 cell.

In Fig. 4C III we observed a perpendicular movement for keratinocyte and HEK293 cells, characterized by high sMM angles (|sMM| > 70°), and similar elongation (0.15 < ε ≤ 0.6). Like in the previous example, this would indicate the cells are migrating in a similar fashion. That, however, is not the case, as turning angles and M.A. dynamics tell us that the migration of these cells as vastly different. All frames of keratinocyte and three frames of HEK293 have low turning angle values, that means movement is performed in the same direction over time and frames of perpendicular displacements are placed one after another in the time sequence. In contrast, many frames of HEK293 are characterised by moderate or high values of turning angles (60° < |α| ≤ 135°). It means, that in preceding frame, HEK293 cell was performing lateral movement (or turn back) and then the direction had to change enough to achieve perpendicular displacement. Here, the values of the turning angle indicate that those frames are placed incidentally between other types of behaviours. A similar situation can be seen in Fig. 4C IV showing the movement askew to the major axis. It is described by moderate values of sMM angles both for keratinocyte and HEK293. Keratinocyte movement again shows low values of turning angle illustrating that askew migration can occur several frames in a row, while the askew movement of HEK293 cell occurs randomly, in-between other types of morphomigrational behaviours, causing the increased values of turning angles. Some higher values of M.A. dynamics (|Δϕ|) characterize the general instability of cell shape, that occurs in several frames of HEK293 cell.

Above examples clearly illustrate that similar morphomigrational behaviours are characterized by similar values of specific building blocks of morphomigrational description, but the occurrence of those behaviours in the context of neighbouring frames, as well as dynamics of cell shape and movement direction, can alter values of the remaining parameters. We expect that further studies will help to better understand morphomigrational behaviours to characterize cell populations.

## Discussion

In this work, we introduce the novel method called *morphomigrational description* that can quantitatively describe cellular behaviour in particular time frame. This description combines two novel parameters (sMM angle and M.A. dynamics) with already existing ones (turning angles and shape elongation) to create four building blocks that describe: displacement regarding cell orientation, dynamics of cell shape, changes of displacement direction and static cell shape, respectively. Those four building blocks are then used for assigning the set of quantitative values for several dynamic behaviours of cells, e.g. migration perpendicular or lateral to the major axis, lateral and perpendicular stretching, tail retraction, turning back along major axis or creating lamellipodium in particular direction regarding a dominant cell shape (Tables 2, 3, Fig. 3, Sup. Figs. S3–S6). The sMM angle is to our knowledge first such parameter that links general shape of the cell with the movement direction, however, a few already existing metrics can be compared with it. For example, some studies focused on substrate topography describe cell shape arrangement on 2D plane^25,29^, but do not link it with migration direction. On the other hand, the predominant migration direction on 2D plane can be represented by the tensor of individual moment of inertia^18^, that is not connected to the cell shape. Moreover some works quantify the dynamics of cellular shape in time. The Fourier shape descriptors and VAR models in TSRVF-PCA space^4,32^ were used for registering the shape and its evolution over time of an experiment. The DECCA parameter^31^ describes the “total amount of cell motion over time” so even non-motile cells with dynamic edges can be characterised by high DECCA values, which was clearly stated by the authors of this work. However, none of the aforementioned methods couple the cell shape with displacement direction, as sMM angle does. Our new parameter gives a novel insight into the analysis of cellular behaviour by linking the cell morphology with displacement direction.

The morphomigrational description bridges the gap between simple verbal descriptions and elaborate mathematical models of cell behaviour. Keratinocyte-like migration is described verbally by a stable morphology and persistent motion^36^, together with a typical fan-shaped appearance^34^, even though the morphology of keratinocyte may vary under different conditions^34,37^. On the other hand, the expected fibroblast morphology in a 2D environment is generally described as ‘elongated’^38^, preferably with large lamellipodium at the front. Those two types of cells serve as archetypes of cellular behaviours and some works directly relate cellular appearance as “fibroblast-like” or “keratocyte-like”^39–42^. Such description uses an intuitive image of a reference model cell to interpret the experimental results, and it seems to be widely understood by the readers. It does not however fulfil the criteria of quantitative description. On the other hand, cell migration and morphology can be described using more sophisticated properties, such as adhesiveness, focal adhesion interactions, occurring forces, contractility^3,38^ or complex mathematical description of cellular shape^37,43^. Morphomigrational description can fill this gap by preserving the quantitative information, all while using simple parameters that can be easily calculated for any sequence of binary images and are intuitive in interpretation.

The great advantage of morphomigrational description over currently existing ones comes from the combination of several quantitative descriptors to analyse the dynamics of migrating cells. Currently existing descriptors present useful, but still fragmentary information, e.g. by calculating movement directionality^15,17^, velocity^8^, predominant migration direction^18^, as well as shape properties^20^ which are mostly averaged over time points or cell population^12^. Our approach uses several descriptors to create a picture of cellular behaviour in specific time point or short time range. Some of those cellular behaviours, such as lateral protrusion and migration (fragments M1 and M4), tail retraction (fragment H3) or migration perpendicular to the major axis (fragment K1) are already identified in the literature as elements of ‘discontinuous’ and “continuous” modes of mesenchymal migration type^44^. However, in that work, cellular activities were identified and classified by visual inspection and the application of ‘behavioural criteria’ that describe cell movement and shape dynamics. These criteria were presented using proper illustrations and intuitive verbal descriptions, while the mathematical relation between the shape orientation and migration direction was not the subject of previous study^44^. In our approach, temporal cell states (morphomigrational behaviours) were elucidated from an image sequence by visual inspection as well, but more importantly, we were able to assign them a set of numerical values of morphomigrational description. The advantages of our description are visible in several cases, e.g. for MEF 3T3 cell in Fig. 2A that migrate laterally to its major axis and then reverses its movement direction. Standard analysis based only on turning angles and cell shape could only show that cell is elongated and reverses its migration direction, while sMM angle additionally indicates that all dynamical actions are performed laterally to the M.A.. In further cases, the morphomigrational approach described lateral or perpendicular stretching (fragments M5 and H4, respectively), while the separate analysis of those fragments could only give an information about rapid changes in movement direction (turning angles) and overall shape change (cell area and elongation). Another such example is the tail retraction identified in fragment H3 which could be only partially described by membrane dynamics^35^ or just a simple change of cell area, while turning angles would show almost no change in the migration direction. In this case, the morphomigrational description provided more detailed description of tail retraction by showing the change of the cell arrangement (M.A. dynamics) combined with rapid change of sMM angle with almost no modification of movement direction (turning angles).

The characteristic cellular morphology and migration mode is reflected in the distributions of quantitative descriptors. Even if the full morphomigrational characterization of cells and cell populations is not the aim of this work, we felt obliged to present patterns created by building blocks of morphomigrational description. In Fig. 4B we have shown that differences between behaviours of exemplary cells are even more visible by plotting sMM angle against turning angle, M.A. dynamics and elongation. Further systematic studies using our approach can be a step forward in biological quantitative analysis, since until now, cell migration was described separately from cellular morphology.

We also identified the specific case of morphomigrational description in which the sMM angle plays a minor role. While most straightforward results come from at least slightly elongated shapes, rounded shapes are more challenging in interpretation. If the fitted ellipse is very close to the circle (indicated by a very low elongation), the major axis, on which sMM angle is constructed, orients itself rather randomly, imposing random value of sMM angle. In such case, the sMM angle does not provide significant information, but even though the rest of building blocks properly present the movement direction and rounded shape.

The sMM angle and M.A. dynamics, similarly to many other metrics calculated between specific time points, are naturally the interval-dependent descriptors^10,15,45,46^. One can imagine that analysing smaller displacements will rather show the plasticity of cellular protrusions, while registering larger displacements will show the dependence between cell geometry and direction of migration. Moreover, in the short time interval the cellular movement is rather diffusive, while in longer time scales the ballistic motion might be observed in general approach^15^. The dependence of sMM angle and M.A. dynamics on time interval is an inherent part of this type of metrics, since sampling is an arbitrary parameter that needs to be optimised based on different environmental conditions, such as: substrate properties, cell type, use of a chemoattractant, etc. This gives room for adaptation of this analysis in different contexts but also requires optimisation of sampling rate. While this issue is beyond the scope of the work presented in this paper, we have provided its brief outlook in the Supplementary Note.

The convolution of cell migration and shape dynamics can also be more sensitive to changing environmental conditions than a separate analysis of cell displacement and shape. Since cell migration and morphology depend on external and internal chemical conditions^47–49^, viral infection^50^, seeding density^51^ or physical microenvironment^49,52^ morphomigrational description will provide better insights into cellular behaviour. Furthermore, we see morphomigrational description become especially useful in studies focused on anisotropic factors modifying cellular behaviour, such as electrotaxis^53^, chemotaxis^4,47,54^ or application of substrate patterning^55^. In all such studies, cells must orient themselves to obtain a particular morphology to migrate in the direction (or opposite to) the stimuli. In our understanding, morphomigrational description might, for example, better determine the moment of stimulation marked by changing morphomigrational parameters in potential moments of stimulation during time sequence.

One of the very first steps in further studies should be the quantification of more morphomigrational behaviours performed on whole cell populations. This matter is not as trivial as it might appear at first glance, since even in a single population of cells we could find several different behaviours that occur simultaneously in time. The identified morphomigrational behaviours will also depend on the migration phases and thus should be considered in the context of the cell dynamics. We have briefly explained this issue in the Supplementary Note. Its main conclusion is, that because of the variability of cell behaviour occurrences the simple averaging of the morphomigrational building blocks is not the right approach to analysing cell populations. Instead, the aim is to analyse the durations of morphomigrational behaviours, find characteristic transitions between behaviours that come sequentially after each other and to define behaviour sequences during migration. Together these will create identifiable patterns which will be specific for cells in the defined conditions. Such an approach can help in a more precise description of dynamical cellular behaviours to understand their functioning. Furthermore, since the morphomigrational states can be described by the set of numbers, it will be possible to organize them in the form of library which in turn can facilitate the process of automatic analysis of cell populations, including deep learning techniques.

## Materials and methods

### Unsigned morphomigrational angle

To better understand the signed morphomigrational angle (*sMM angle* or *sMM*), we introduce the initial calculation procedure of simpler, unsigned morphomigrational angle (*uMM* angle or *uMM*). It informs us in which direction the cell centroid moves regarding the major axis (M.A.) of the shape. The uMM angle is an acute angle constructed on the M.A. of an ellipse fitted to the binary shape and displacement vector between current (n) and following (n + 1) frame as shown in Fig. 1B. The procedure of ellipse fitting is described in further sections of “Materials and methods”. For clarity of further descriptions, we use the shorter phrase: “major axis of shape” which means “major axis of an ellipse fitted to the binary mask of the shape”.

The uMM angle is always an acute angle, so it may be constructed on right or left side of displacement vector. Figure 1C presents this situation on the very same keratinocyte registered at different time points. In the top row the keratinocyte turns slightly to the right and uMM angle is constructed on the right side of displacement vector, while in the bottom row keratinocyte turns slightly to the left, having uMM angle on the left side of displacement vector. However, in both cases the value of uMM angle is very similar: 71° and 70° respectively. Thus, the uMM angle does not inform about turning direction of the cell (which is measured by turning angle), but links the spatial arrangement of the shape with its movement direction. Nonetheless, it is easy to imagine four different directions of displacement vectors (pointing to the right and left side of the image, both above and below major axis) resulting in four very similar values of unsigned uMM angle, but still describing four different morphomigrational behaviours. To clarify this ambiguous situation we calculate the signed morphomigrational angle (sMM angle).

### Signed morphomigrational angle and M.A. dynamics

The uMM angle may be susceptible to a variety of interpretations, which might be confusing when drawing conclusions about cell morphology and movement. Hence, to get more detailed information, we track whether the uMM angle is always constructed on the same side of major axis or not. To achieve that, we assign the positive sign to sMM angle if uMM is constructed on the same side of M.A. as in the first frame and negative sign if uMM angle is constructed on the other side (Fig. 1D). The detailed algorithm for calculating sMM angle is presented in Sup. Methods S1 and Sup. Fig. S2.

Using this procedure we also define the angle between major axes (M.A.) in two consecutive time frames (*n, n* + *1*), calling it M.A. dynamics and denoting with *Δϕ*. Low values of M.A. dynamics inform about similar shape orientation on 2D plane in two consecutive time frames, while large values can describe rapid changes in cell shape or rounded shapes as mentioned in further sections.

### Image analysis and quantitative descriptors of cell shape and cell migration

First, separate cells were cropped from the time-lapse image sequence. Only migration of single cells that were not in contact with other ones for at least 80% of total experiment time was analysed. The algorithm works on binary masks, that in our case were segmented manually using FIJI software. However, it is also possible to use any automatic software for image segmentation as long as it gives the binary masks as the output, although these procedures were not in the scope of current work. Cell masks were analysed using custom code written in Matlab 2016a (Math Works Inc.) that works as follows: the binary shape properties were obtained using Matlab function *regionprops*. The displacement vector for each frame was defined as the distance between the centroids in currently analysed (*n*) and following (*n* + *1*) frame. Turning angle (*α*) was calculated between preceding displacement vector (between *n* − *1* and *n* frames) and current displacement vector (between *n* and *n* + *1* frames), where clockwise turns were marked with positive sign. Shape elongation (*ε*) is calculated as *ε* = *1* − *a/b* where *a* and *b* denote minor and major axis of ellipse fitted to the analysed shape, respectively. Ellipse fitting was performed by Matlab built-in function *regionprops* that fits the ellipse of the same second-moments as the analysed binary shape. Elongation *ε* = *0* signifies a perfectly round shape and *ε* = *1* describes an infinitely elongated ellipse. The major axis (M.A.) orientation regarding X axis was obtained using *regionprops* function as well. Consecutive steps of calculation sMM angle are presented visually in Sup. Fig. S2.

To present the proof of principle of morphomigrational description, we have chosen and carefully analysed three individual cells, each one of different cell type (fish keratinocyte, MEF3T3 and HEK293). Different cell types were chosen to represent distinct types of cellular behaviours, to depict them using quantitative descriptors. Time intervals for analysis were chosen to show the scheme of morphomigrational description and were depended on individual cell velocity: for keratinocyte 20 s (91 frames), HEK293 cell: 4 min (76 frames), MEF 3T3: 10 min (49 frames).

### Cell culture and time-lapse experiments

The study was performed in line with ARRIVE guidelines. All method/experimental protocol in the study was assessed by the Local Ethical Commission for Investigations on Animals in Poznań as not invasive on the level requiring acceptance of the Committee. All procedures on live animals were carried out in strict accordance with the Act on the Protection of Animals Used for Scientific or Educational Purposes in Poland, according to which the permission of The Local Ethical Commission for Investigations on Animals in Poznań at Poznań University of Life Sciences was not needed. All members of the research staff were trained in animal care, handling, and euthanasia by Polish Association of Laboratory Animal Science (PolLASA). Live fish, golden molly (*Poecilia sphenops*) were maintained in Laboratory of Inland Fisheries and Aquaculture (Department of Zoology) of Poznań University of Life Sciences (Unit no. 0091, registered by National Ethics Commission (Warsaw, Poland). Fish were euthanized with an overdose of tricaine methanesulfonate (MS222, 300 mg L^−1^) by prolonged immersion, then scales were collected post mortem. Fish epithelial keratinocytes were collected according to standard procedures^56^. After being collected from the fish, the scales were placed for 15 min in cell medium composed of DMEM High Glucose (BioWest) with 10% Fetal Bovine Serum (Gibco) + 1% Penicillin/Streptomycin (Bio West). Then, each scale was placed in a separate 35 mm glass bottom plate (CellVis) in 20 µl droplet of medium and covered with a round 18 mm coverslip. A few droplets of sterile distilled water were placed inside the dish to prevent the sample from drying out. Then glass-bottom dishes were sealed with parafilm for 24 h incubation in room temperature. During the incubation, a large cluster of keratinocytes migrated from the scale onto the glass substrate. After the incubation, glass-bottom dishes were gently filled with cell media to separate top coverslip from the dish. The samples were left for 2 h to allow the cells to recover after the coverslip separation. The samples were washed gently with Phosphate Buffered Saline (PBS) without Ca^2+^ and Mg^2+^ and left with fresh PBS for the next 40 min to partially disperse the cell group and obtain separately migrating keratinocytes. Subsequently, PBS was gently replaced with cell medium, and the samples were incubated for the next 1.5 h, which was sufficient to achieve a single cell migration behaviour similar to that before incubation with PBS. After this procedure keratinocytes were ready for imaging.

MEF 3T3 cells were cultured in medium composed of DMEM Low Glucose Medium (DMEM LG—Bio West) supplemented with 10% FBS (Gibco) and 1% PS (Bio West). For the experiment, the fibroblasts were seeded at a concentration of 12,000–15,000 on the 35 mm glass bottom dish (CellVis) and incubated for 10 h in a cell incubator. After this, MEF 3T3 cells were ready for imaging.

HEK293 cells were cultured in DMEM HG (Bio West) supplemented with 10% FBS (Gibco) and 1% PS (Bio West). For the experiment, cells were seeded at a concentration of ~ 100,000 cells in a 35 mm glass-bottom dish (CellVis) and incubated for 18 h in cell incubator. After this procedure, HEK293 cells were ready for imaging.

Time-lapse experiments were performed on Zeiss Axio Observer Z1 inverted microscope equipped with 10x/0.5 NA dry objective and Hamamatsu Orca 4.0 V2 camera. Keratinocytes were kept at room temperature and atmosphere, MEF3T3 and HEK293 cells were kept in 37 °C and 5% CO_2_. Keratinocyte migration was observed for 30 min in a 10-s interval, MEF 3T3 for 9 h with a 2.5 min interval and HEK293 cells for 5 h with a 2-min interval.

## Supplementary Information

Supplementary Information 1.

Supplementary Information 2.

## Data Availability

The datasets generated during and/or analyzed during the current study are available from the corresponding author on reasonable request.
