# Supplementary material for: Morphomigrational description as a new approach connecting cell's migration with its morphology
Source: Sci Rep. 2023 Jul 7;13:11006. doi: 10.1038/s41598-023-35827-9 (PMC10328925; doi:10.1038/s41598-023-35827-9)
Supplement: Supplementary file 1 — Supplementary Information 1. [file 41598_2023_35827_MOESM1_ESM.docx]

**Supplementary Information File for**

“Morphomigrational description as a new approach connecting cell’s migration with its morphology”
by Tomasz Kołodziej, Aleksandra Mielnicka, Daniel Dziob, Anna Katarzyna Chojnacka,
Mateusz Rawski, Jan Mazurkiewicz and Zenon Rajfur.

**Supplementary Method S1:** Algorithm for calculating sMM angle.

The proper identification of M.A. sides, thus the sign of sMM angle requires tracking the cell shape along the entire time sequence. To properly explain the procedure, we need to mention that coordinate system of analysed image always starts in its upper-left corner which is imposed simply by the numeration of rows and columns. The procedure is presented visually in Supplementary Figure S2. It starts from calculating uMM angle (steps 1-4) and is followed by rotation of resulting image to horizontal position of major axis (step 5). Because of that, the uMM angle in rotated image is always placed above or below of major axis. First location of uMM angle determines the positive sign in particular image sequence, regardless whether it is above or below rotated M.A., while the opposite side is marked with negative sign. In the first frame the image is always rotated clockwise about lower end of major axis (higher Y-axis value). In the next frames, binary masks and displacements vectors are rotated to find the sMM angle sign as well, but the point of rotation as well as rotation angle in each frame are chosen to minimize the differences between rotation angles ϕ in consecutive frames. Thus, except the first frame, the rotation angle does not need to be always clockwise and point of rotation does not need to be at the lower end of major axis. This procedure allows for proper calculation of sMM angle during the shape rearrangement in time. The proper identification of rotation points and thus, all sides of major axis is presented in Supplementary Figure S2.

**Supplementary Figure S2:** Graphic presentation of algorithm for sMM angle calculation presented for two cases of migrating cells:

1. Fish keratinocyte analysed every 100 s to better visualise binary masks in consecutive frames. The sMM sign is positive along the whole sequence. In two first frames however, in STEP 5 the shape is rotated clockwise around the lower end of M.A., while in third and fourth frames the shape rotation occurs counterclockwise around the higher end of M.A. due to the repositioning of cell shape. Due to this procedure the sign of sMM angle is calculated properly, since here the displacement vector constantly points to the same side of M.A.
2. MEF 3T3 fibroblast migrating along its major axis, analysed every 20 minutes to better visualise binary masks in consecutive frames. The sMM sign changes in the third frame since the displacement vector points to the other side of M.A. than in the first frame.

Green masks and blue outlines represent currently analysed shape and following shape, respectively. Black arrow signifies movement direction (magnified displacement vector), green line is major axis of an ellipse fitted to current shape (green ellipse). Yellow and blue circles mark shape centroids in current (n) and following (n+1) frame, respectively. Red area with stripes marks uMM angle. Purple point marks rotation end of major axis, purple arrow points into direction of rotation. Scale bar represents 20 micrometres.


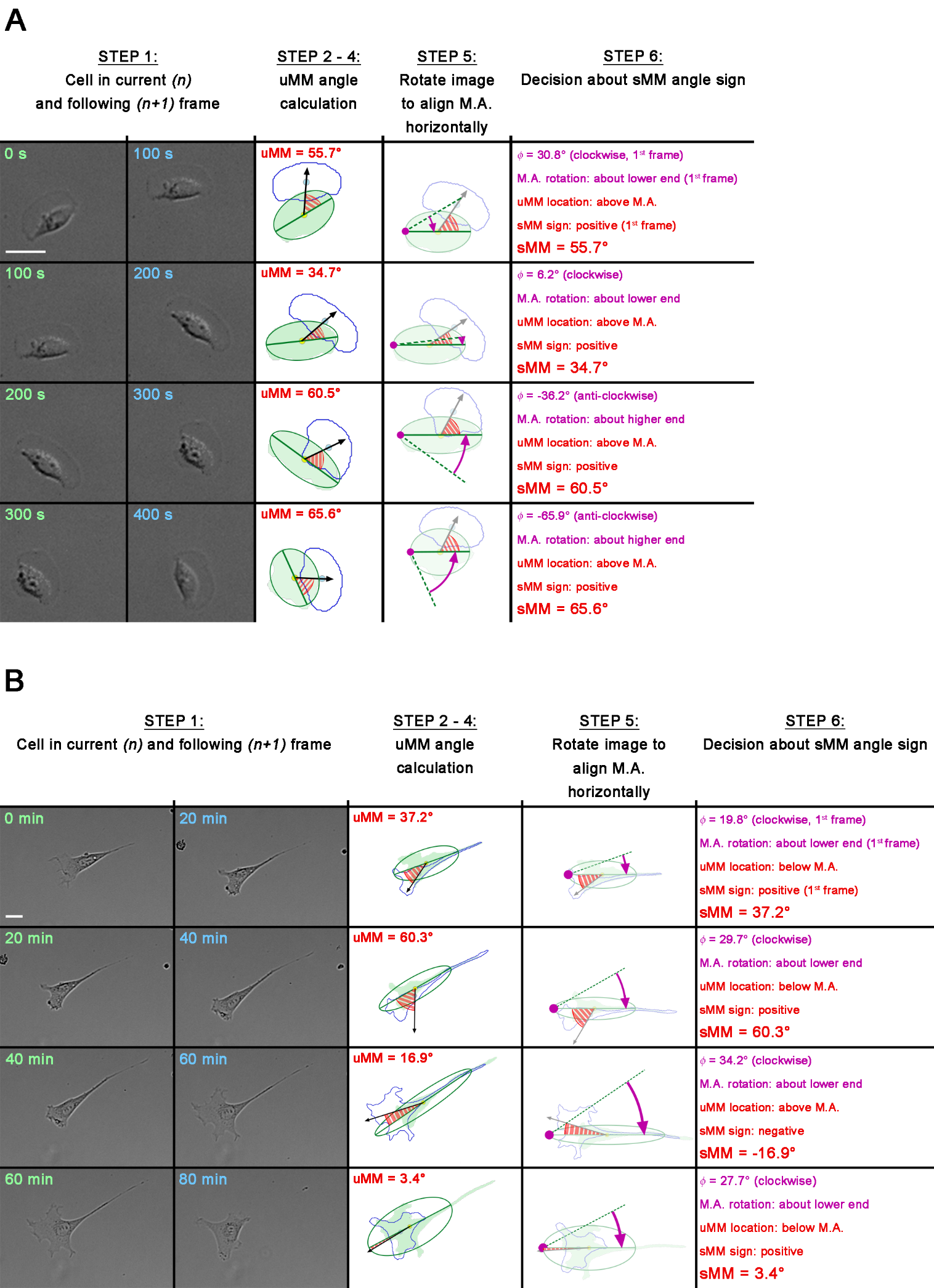


**Supplementary Figure S3:** Illustration of sMM angle, turning angle and M.A. dynamics of fragments K1, H1, H2 & H3. Scale bar represents 20 micrometres.


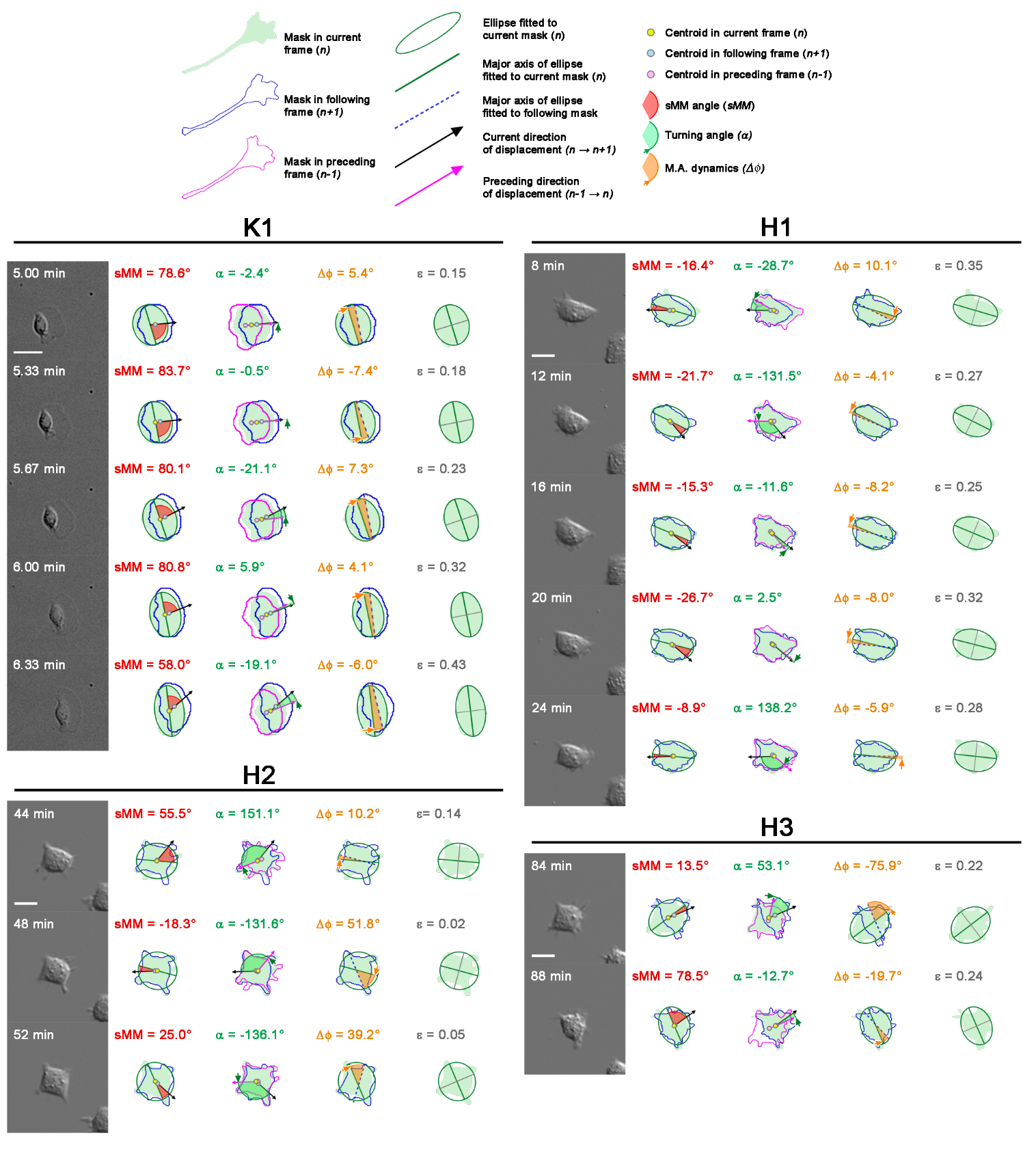


**Supplementary Figure S4:** Illustration of sMM angle, turning angle and M.A. dynamics of fragments H4, H5 & M1. Scale bar represents 20 micrometres
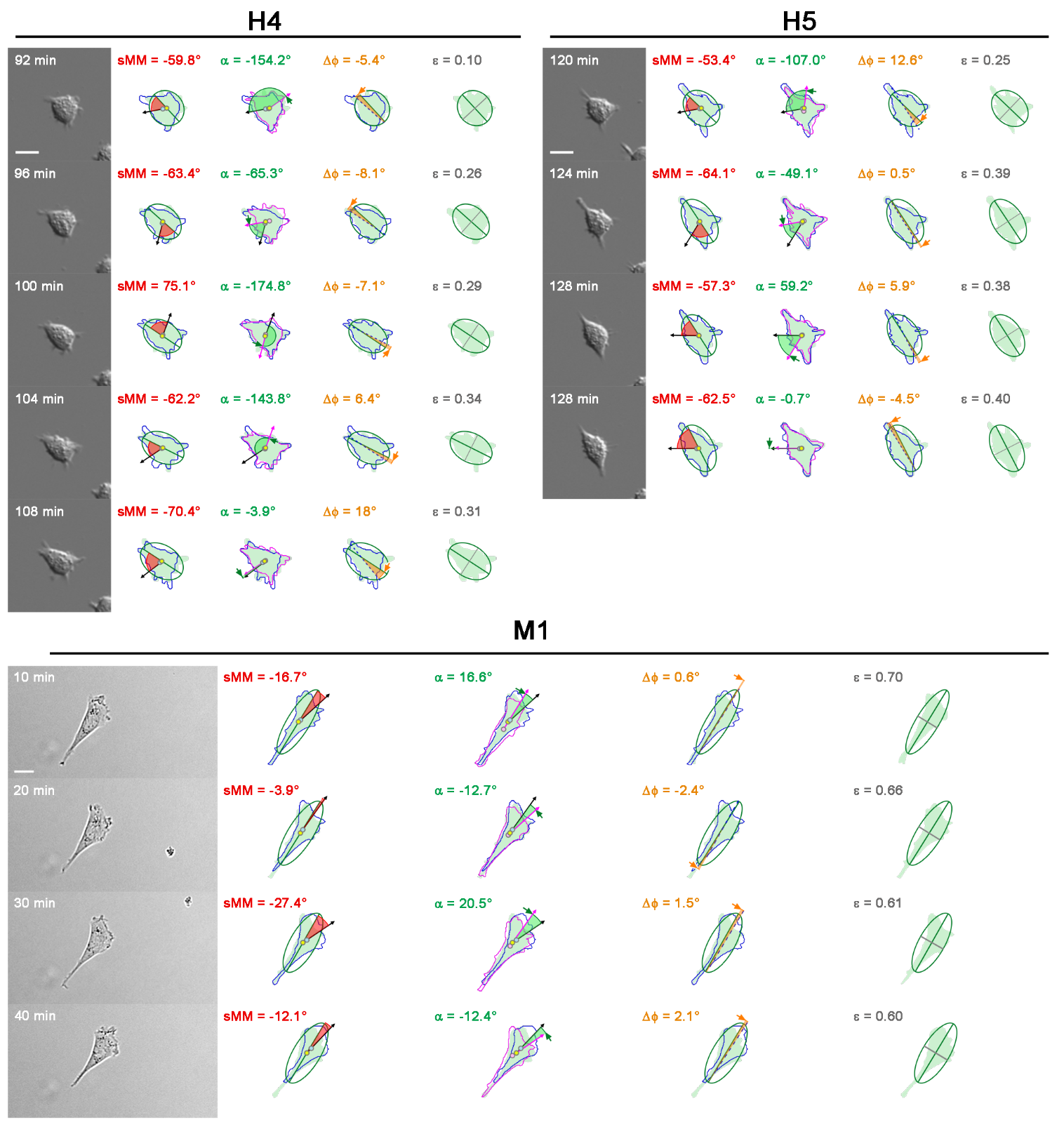


**Supplementary Figure S5:** Illustration of sMM angle, turning angle and M.A. dynamics of fragments M2 & M3.
Scale bar represents 20 micrometres


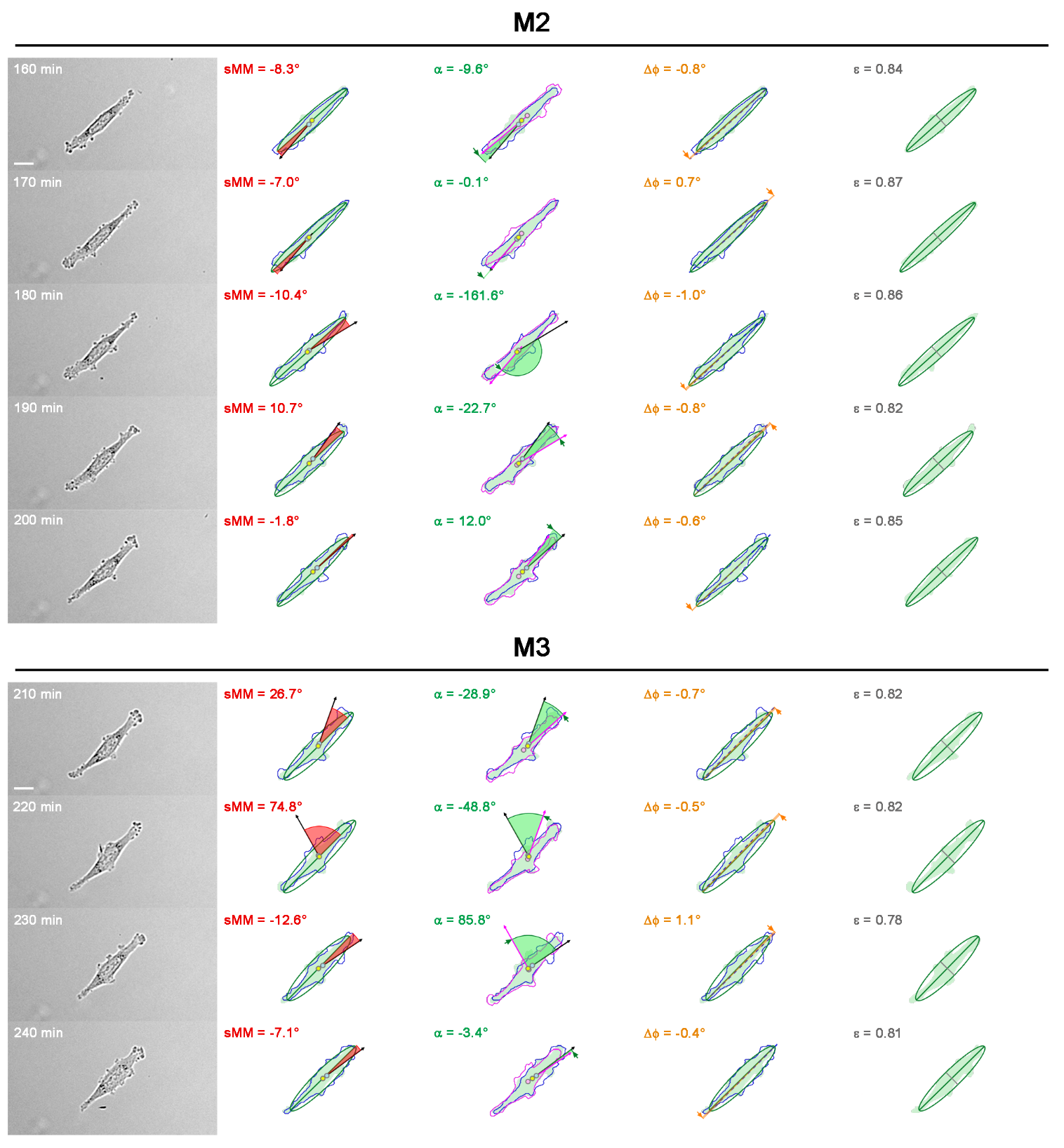


**Supplementary Figure S6:** Illustration of sMM angle, turning angle and M.A. dynamics of fragments M4 & M5.
Scale bar represents 20 micrometres

**
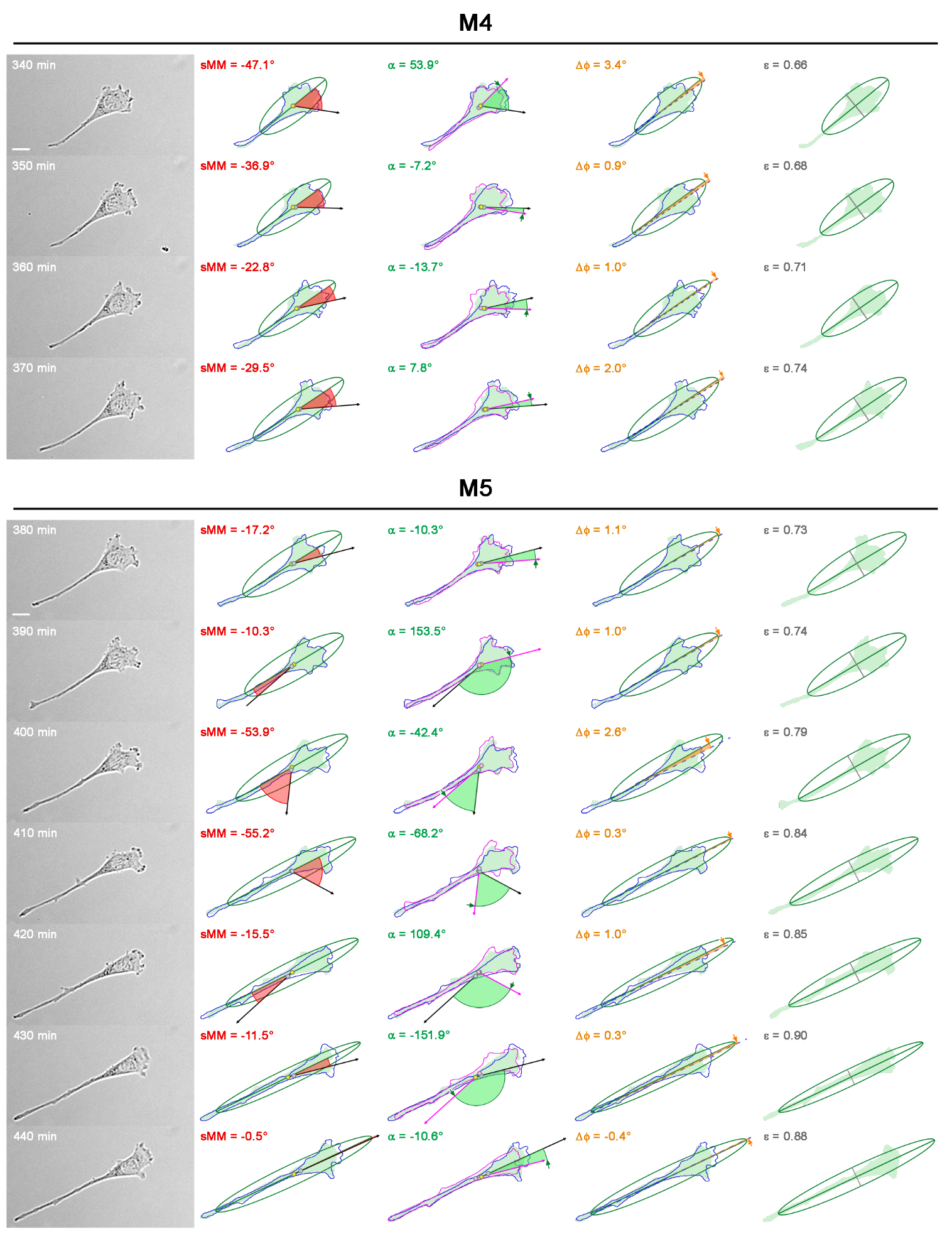
**

**Supplementary Table S7:** Exact values and coarse-grain ranges of quantitative descriptors presented in Figure 3 and Tables 2-3.

| Symbol | Frame | Time [min] | sMM | | α | | Δϕ | | ε | |
| --- | --- | --- | --- | --- | --- | --- | --- | --- | --- | --- |
|  |  |  | exact [deg] | Coarse-grain | exact [deg] | Coarse-grain | exact [deg] | Coarse-grain | exact [norm] | Coarse-grain |
| K1 | 16 | 5.0 | 78.6 | high | -2.4 | low | 5.4 | very low | 0.15 | moderate |
|  | 17 | 5.3 | 83.7 | high | -0.5 | low | -7.4 | very low | 0.18 | moderate |
|  | 18 | 5.7 | 80.1 | high | -21.1 | low | 7.3 | very low | 0.23 | moderate |
|  | 19 | 6.0 | 80.8 | high | 5.9 | low | 4.1 | very low | 0.32 | moderate |
|  | 20 | 6.3 | 58.0 | moderate | -19.1 | low | -6.0 | very low | 0.43 | moderate |
| H1 | 3 | 8 | -16.4 | low | -28.7 | low | 10.1 | low | 0.35 | moderate |
|  | 4 | 12 | -21.7 | low | -131.5 | high | -4.1 | very low | 0.27 | moderate |
|  | 5 | 16 | -15.3 | low | -11.6 | low | -8.2 | very low | 0.25 | moderate |
|  | 6 | 20 | -26.7 | low | 2.5 | low | -8.0 | very low | 0.32 | moderate |
|  | 7 | 24 | -8.9 | very low | 138.2 | very high | -5.9 | very low | 0.28 | moderate |
| H2 | 12 | 44 | 55.5 | moderate | 151.1 | very high | 10.2 | low | 0.14 | moderate |
|  | 13 | 48 | -18.3 | low | -131.6 | high | 51.8 | high | 0.02 | low |
|  | 14 | 52 | 25.0 | low | -136.1 | very high | 39.2 | moderate | 0.05 | low |
| H3 | 22 | 84 | 13.5 | very low | 53.1 | low | -75.9 | very high | 0.22 | moderate |
|  | 23 | 88 | 78.5 | high | -12.7 | low | -19.7 | low | 0.24 | moderate |
| H4 | 24 | 92 | -59.8 | moderate | -154.2 | very high | -5.4 | very low | 0.10 | low |
|  | 25 | 96 | -63.4 | moderate | -65.3 | moderate | -8.1 | very low | 0.26 | moderate |
|  | 26 | 100 | 75.1 | high | -174.8 | very high | -7.1 | very low | 0.29 | moderate |
|  | 27 | 104 | -62.2 | moderate | -143.8 | very high | 6.4 | very low | 0.34 | moderate |
|  | 28 | 108 | -70.4 | high | -3.9 | low | 18 | low | 0.31 | moderate |
| H5 | 31 | 120 | -53.4 | moderate | -107.0 | high | 12.6 | low | 0.25 | moderate |
|  | 32 | 124 | -64.1 | moderate | -49.1 | low | 0.5 | very low | 0.39 | moderate |
|  | 33 | 128 | -57.3 | moderate | 59.2 | low | 5.9 | very low | 0.38 | moderate |
|  | 34 | 132 | -62.5 | moderate | -0.7 | low | -4.5 | very low | 0.4 | moderate |
| M1 | 2 | 10 | -16.7 | low | 16.6 | low | 0.6 | very low | 0.70 | high |
|  | 3 | 20 | -3.9 | very low | -12.7 | low | -2.4 | very low | 0.66 | high |
|  | 4 | 30 | -27.4 | low | 20.5 | low | 1.5 | very low | 0.61 | high |
|  | 5 | 40 | -12.1 | very low | -12.4 | low | 2.1 | very low | 0.60 | moderate |
| M2 | 17 | 160 | -8.3 | very low | -9.6 | low | -0.8 | very low | 0.84 | high |
|  | 18 | 170 | -7.0 | very low | -0.1 | low | 0.7 | very low | 0.87 | high |
|  | 19 | 180 | -10.4 | very low | -161.6 | very high | -1.0 | very low | 0.86 | high |
|  | 20 | 190 | 10.7 | very low | -22.7 | low | -0.8 | very low | 0.82 | high |
|  | 21 | 200 | -1.8 | very low | 12 | low | -0.6 | very low | 0.85 | high |
| M3 | 22 | 210 | 26.7 | low | -28.9 | low | -0.7 | very low | 0.82 | high |
|  | 23 | 220 | 74.8 | high | -48.8 | low | -0.5 | very low | 0.82 | high |
|  | 24 | 230 | -12.6 | very low | 85.8 | moderate | 1.1 | very low | 0.78 | high |
|  | 25 | 240 | -7.1 | very low | -3.4 | low | -0.4 | very low | 0.81 | high |
| M4 | 35 | 340 | -47.1 | moderate | 53.9 | low | 3.4 | very low | 0.66 | high |
|  | 36 | 350 | -36.9 | low | -7.2 | low | 0.9 | very low | 0.68 | high |
|  | 37 | 360 | -22.8 | low | -13.7 | low | 1.0 | very low | 0.71 | high |
|  | 38 | 370 | -29.5 | low | 7.8 | low | 2.0 | very low | 0.74 | high |
| M5 | 39 | 380 | -17.2 | low | -10.3 | low | 1.1 | very low | 0.73 | high |
|  | 40 | 390 | -10.3 | very low | 153.5 | very high | 1.0 | very low | 0.74 | high |
|  | 41 | 400 | -53.9 | moderate | -42.4 | low | 2.6 | very low | 0.79 | high |
|  | 42 | 410 | -55.2 | moderate | -68.2 | moderate | 0.3 | very low | 0.84 | high |
|  | 43 | 420 | -15.5 | low | 109.4 | high | 1.0 | very low | 0.85 | high |
|  | 44 | 430 | -11.5 | very low | -151.9 | very high | 0.3 | very low | 0.90 | high |
|  | 45 | 440 | -0.5 | very low | -10.6 | low | -0.4 | very low | 0.88 | high |
