# Supplementary material for: Morphomigrational description as a new approach connecting cell's migration with its morphology
Source: Sci Rep. 2023 Jul 7;13:11006. doi: 10.1038/s41598-023-35827-9 (PMC10328925; doi:10.1038/s41598-023-35827-9)
Supplement: Supplementary file 2 — Supplementary Information 2. [file 41598_2023_35827_MOESM2_ESM.docx]

**Supplementary Note for**

“Morphomigrational description as a new approach connecting cell’s migration with its morphology”
by Tomasz Kołodziej, Aleksandra Mielnicka, Daniel Dziob, Anna Katarzyna Chojnacka,
Mateusz Rawski, Jan Mazurkiewicz and Zenon Rajfur.

1. **Comment on of the morphomigrational characterization of subpopulations.**

To test the morphomigrational description, three different types of cells were selected for the analysis. These three cell lines were chosen precisely because, as widely reported in literature, they display different migration patterns consisting of various cellular behaviours. The further quantification of whole cellular populations seems to be an obvious next step in application of morphomigrational description. However, while this subject is out of scope of this work, we would like to briefly consider the complexity of such quantification using only three separate cells from each examined type (3 keratinocytes, 3 MEF 3T3 cells and 3 HEK 293 cells).

The morphomigrational description of the whole cellular population would be the easiest for keratinocytes. The Sup. Note. Fig. 1. shows three different keratinocytes and the illustration of their quantitative descriptors. One can see that the values of the 3 parameters in the graphs are reasonably consistent between the 3 cells, even though you can see that cell #1 has slightly lower values of elongation than other 2 cells. In this case the morphomigrational analysis of the whole population would be easy.

*
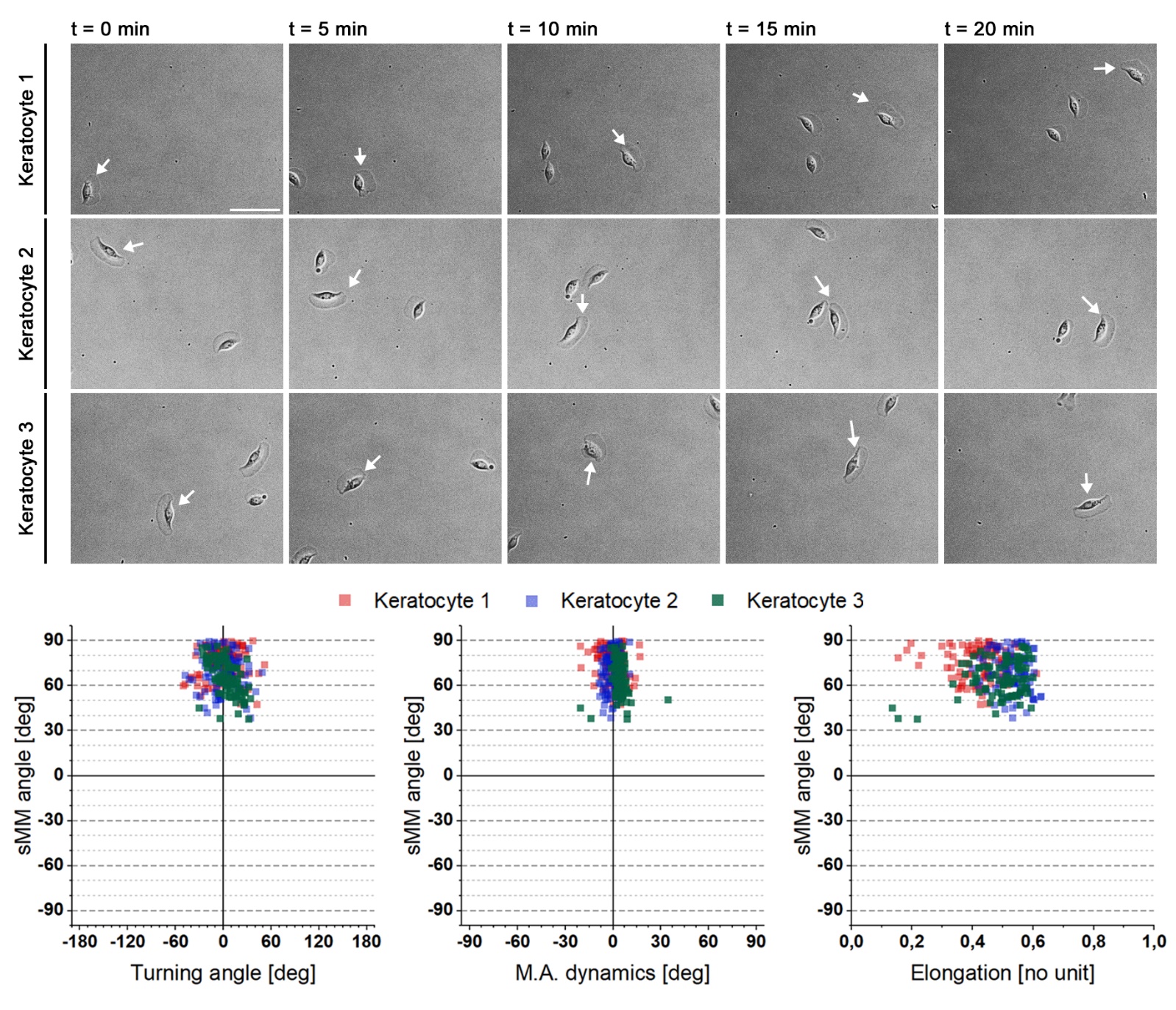
*

*Sup. Note. Fig. 1. Three exemplary fish epithelial keratinocytes from the same experiment described by plots of their morphomigrational descriptors, similarly to Fig. 4B in the manuscript. Scale bar represents 50 µm.*

The matter becomes more complex for HEK293 cells, even if they come from the very same experiment. From the figure below, it is clear that while sMM vs turning angle is evenly scattered for all 3 cells, one cell (#2 green) has much lower values of M.A. dynamics and is significantly more elongated than the remaining two. Those differences are caused by different proportions of morphomigrational behaviours expressed by three separate cells.


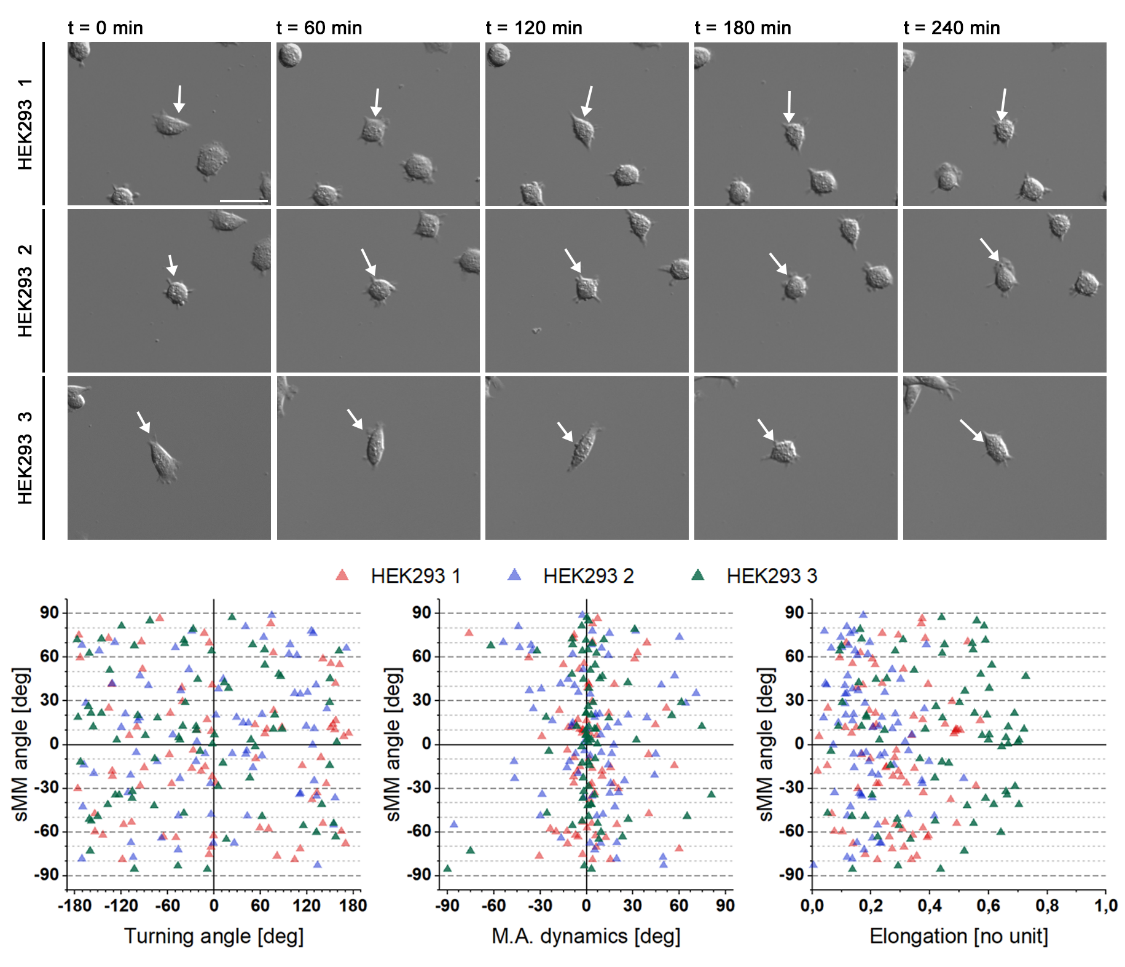


*Sup. Note. Fig. 2. Three exemplary HEK293 cells from the same experiment, described by plots of their morphomigrational descriptors, similarly to Fig. 4B in the manuscript. Scale bar represents 50 µm.*

The morphomigrational description of the whole population gets even more complicated for MEF 3T3 cells. Those cells exhibit one of the best described modes of cell migration: mesenchymal type which incorporates the following steps: 1) protrusion extension, 2) adhesion, 3) body translocation and 4) rear retraction. Each of this step carry a shape change which will result in different set of morphomigrational descriptors. If we were to simply average this, these behaviours would be lost, and the resulting metric would not give us any information about the cells. In the figure below you can see that 3 MEF3T3 cells and the differences in cell behaviours are very clear between these 3 individuals. Averaging value spread like this would be meaningless.


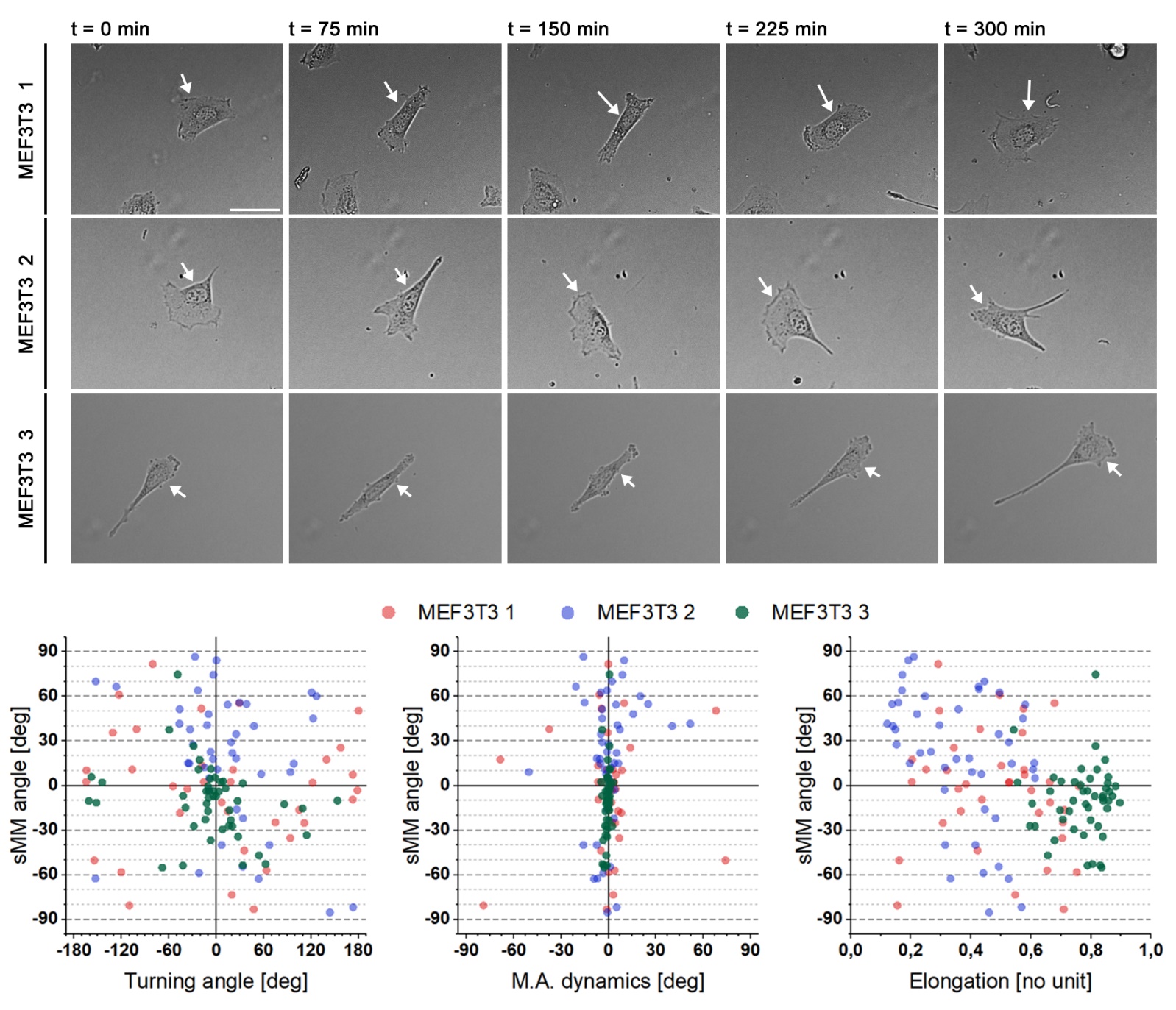


*Sup. Note. Fig. 3. Three exemplary MEF3T3 cells from the same experiment, described by plots of their morphomigrational descriptors, similarly to Fig. 4B in the manuscript. Scalebar represents 50 µm.*

These examples of population behaviours we discussed above lead us to the next point.

Robust population analysis is not a trivial question that can be done simply by using 3 cells of each cell type and averaging their behaviours. Here are the aspects of population analysis that we consider to be crucial for the systematic study of cellular populations:

1. Creating a „library” of morphomigrational behaviors of different cells, with their proper quantification
2. Determining the proper timescales of different behaviors within each population
3. Detection and description of behavior sequences (antecedence and precedence of behaviors or in other words detecting time patterns of behaviors that come before or after another) per population
4. Problem of data sampling and variability in observable behaviors within population, issue of invariability to sampling, creating guidelines for sampling for use with this method per population analysis, on which we will elaborate more, in the answer to the 2^nd^ question of the reviewer.

All the above-mentioned issues are out of scope of this work, and we expect them to be elaborated in further studies.

1. **Comment on the selection of sampling interval in the morphomigrational description.**

As mentioned in the discussion, not unlike most if not all the existing cell migration descriptors, our method
is ***not invariable*** to sampling times. This is not a detriment of our method, but simply inherent nature of any quantification of migration which relies on time-lapse data analysis. Indeed, sampling time selection is a well know issue within the scientific community working on migration analysis. While ***not invariable to sampling interval***, our metrics are ***invariable to type of cellular behaviours*** for which the morphomigrational description was designed. We would like to briefly illustrate the problem of the sampling interval choice by showing the differences in detected cell behaviours in the figures below. This by no means is exhaustive in considering impact of sampling on analysing the results, but it may help the reader understand how different sampling times can influence the observed morphomigrational behaviours and thus how it influences the quantified parameters.

- In the examples below for keratinocyte, the sampling time changes do not impact its morphomigrational behaviours, rather the frequency at which these behaviours are observed. The sMM are consistently high value and turning angles are small. These are simply because unstimulated keratinocytes migrate relatively “straight” and perpendicularly to its major axis.


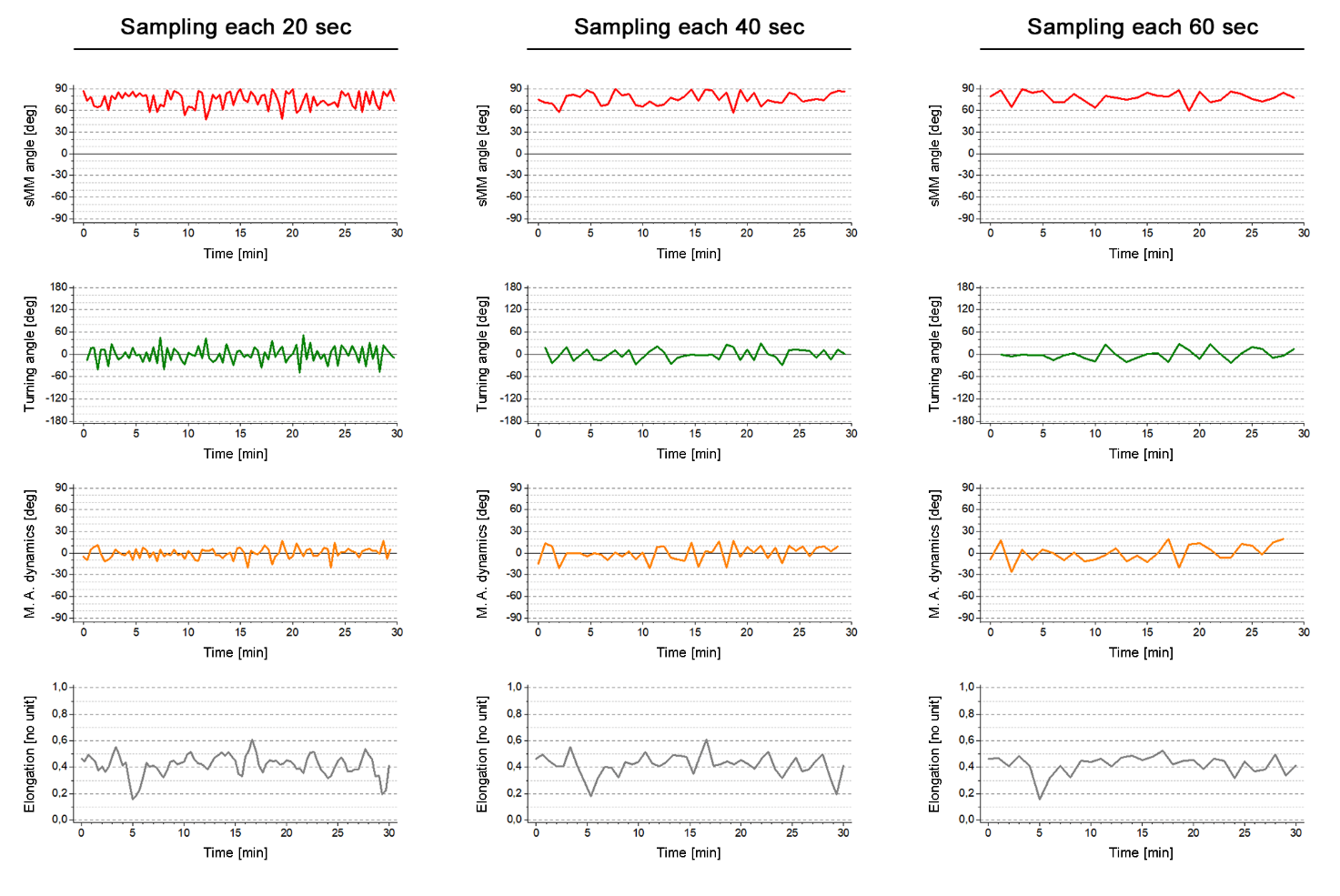


*Sup. Note. Fig. 4. Three different sampling of fish epithelial keratinocyte, that is analysed in the manuscript.*

- The changes in sampling times are more complex when one analyses the dynamics of HEK293 cell. Here, the sMM angle sign changes more frequently and has high values within the 30-50 min interval when sampling time is 4 min. This points to lateral and askew stretching on both sides of the major axis. This granular information is lost when the sampling time is extended to 8 minutes within the same time frame. Similarly, M.A. dynamics is affected by extending sampling times. Looking at the time frame of 50-100 minutes, sampling with 4-minute interval shows lower M.A. dynamics suggesting the cell is not changing its geometric orientation. However, 12 minutes sampling interval shows higher M.A. dynamics, which points to more rapid changes in cell orientation. While overall the movement of HEK293 cell is classified as chaotic, when considering shorter vs longer sampling times, we can observe different granular components of this movement.


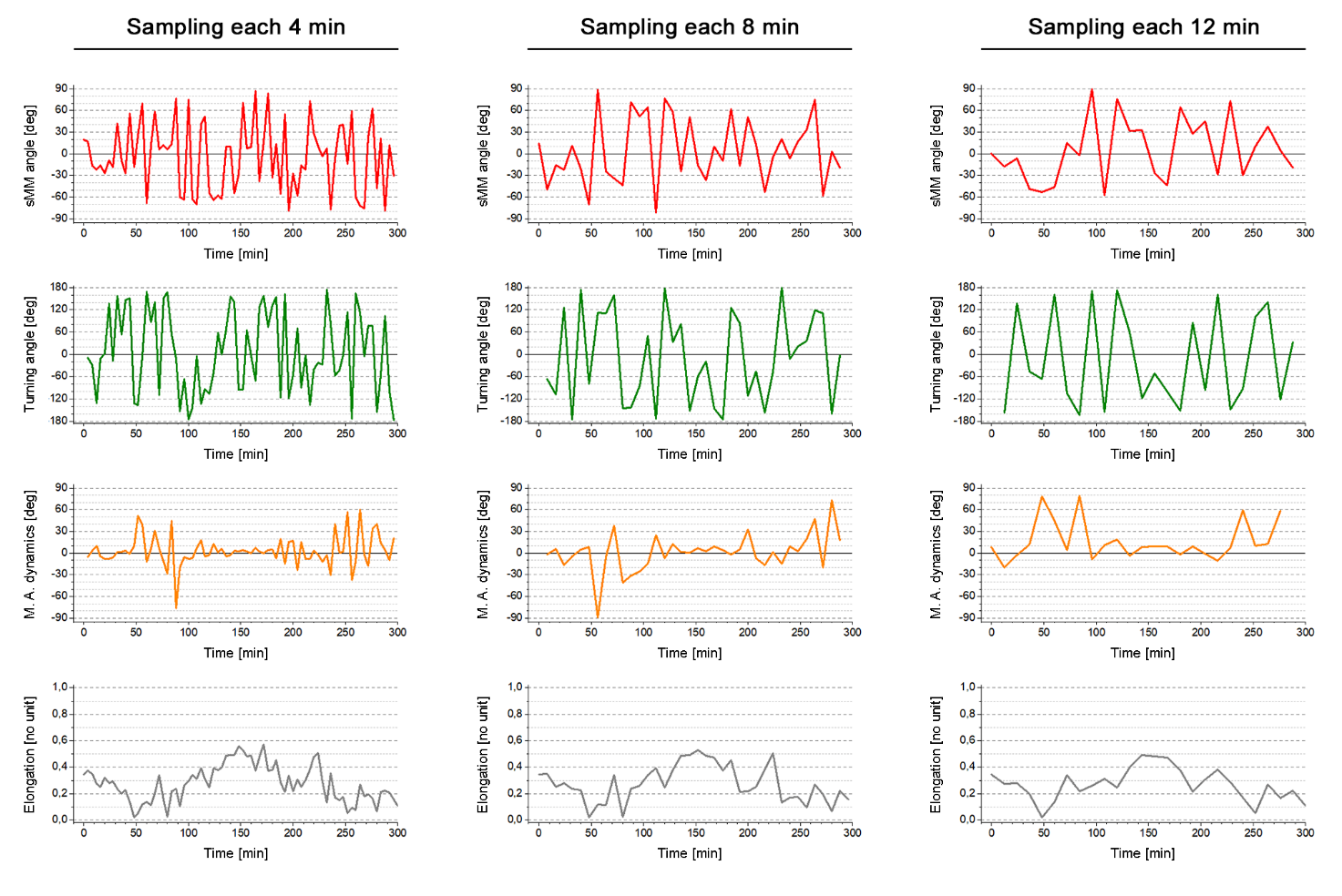


*Sup. Note. Fig. 5. Three different sampling of HEK293 cell, that is analysed in the manuscript.*

- For MEF cell, the sampling rate changes make the most difference. Frequent sampling times (10 min) show lower values of sMM angle indicate a parallel movement, while extending the sampling times results in detection of movements that are “less parallel” to its major axis. By changing sampling times, we observe different types of movement altogether. Same case is for turning angle, where using shorter sampling intervals reveal additional fragments of parallel movements, that are visible between incidental U-turns, while longer sampling time again results in this granular information being lost. For this cell, values of M.A. dynamics don’t change as the cell exhibits stability in 2D plane. However, this would likely not be the case for other MEF like we illustrated previously. This cell type can change its shape during different phases of its migration and within population variability is predictably high.


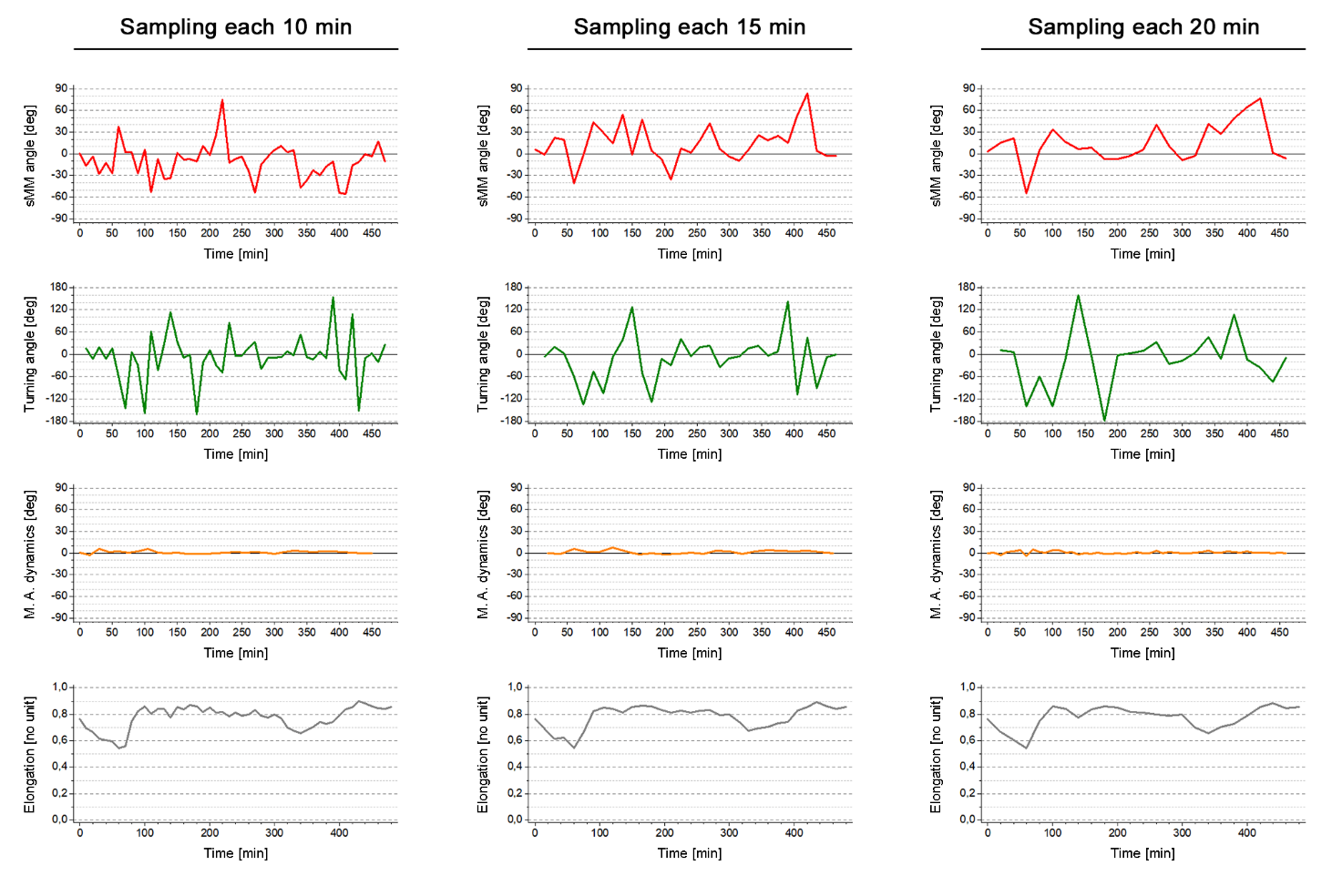


*Sup. Note. Fig. 6. Three different sampling of MEF3T3 cell, that is analysed in the manuscript.*

The above analysis is by no means an exhaustive explanation of the issue of data sampling. However, by showing these exemplary figures we aim to present the reader the brief outline of the problem of data sampling in the morphomigrational description and show the possible ways of its further development.
